# Highly Oriented Direct-Spun Carbon Nanotube Textiles Aligned by In Situ Radio-Frequency Fields

**DOI:** 10.1021/acsnano.2c02875

**Published:** 2022-05-31

**Authors:** Liron Issman, Philipp A. Kloza, Jeronimo Terrones Portas, Brian Collins, Afshin Pendashteh, Martin Pick, Juan J. Vilatela, James A. Elliott, Adam Boies

**Affiliations:** † Department of Engineering, University of Cambridge, Cambridge CB2 1PZ, United Kingdom; ‡ Department of Materials Science & Metallurgy, University of Cambridge, Cambridge CB3 0FS, United Kingdom; § BSC Associates Ltd, 2 Pilgrims Way, Ely CB6 3DL, Cambridgeshire, U.K.; ∥ IMDEA Materials Institute, c/Eric Kandel 2, Getafe 28906, Madrid Spain; ⊥ Q-Flo Limited, Buckhurst House, 42/44 Buckhurst Avenue, Sevenoaks TN13 1LZ, United Kingdom

**Keywords:** alignment, carbon nanotubes, high voltage, radio frequency, Lorentz force, aerosols

## Abstract

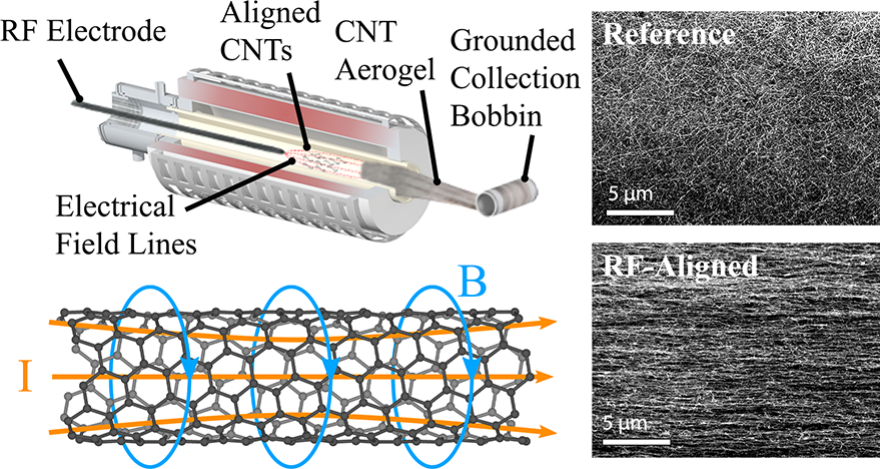

Carbon nanotubes (CNTs) individually exhibit exceptional physical properties, surpassing state-of-the-art bulk materials, but are used commercially primarily as additives rather than as a standalone macroscopic product. This limited use of bulk CNT materials results from the inability to harness the superb nanoscale properties of individual CNTs into macroscopic materials. CNT alignment within a textile has been proven as a critical contributor to narrow this gap. Here, we report the development of an altered direct CNT spinning method based on the floating catalyst chemical vapor deposition process, which directly interacts with the self-assembly of the CNT bundles in the gas phase. The setup is designed to apply an AC electric field to continuously align the CNTs in situ during the formation of CNT bundles and subsequent aerogel. A mesoscale CNT model developed to simulate the alignment process has shed light on the need to employ AC rather than DC fields based on a CNT stiffening effect (z-pinch) induced by a Lorentz force. The AC-aligned synthesis enables a means to control CNT bundle diameters, which broadened from 16 to 25 nm. The resulting bulk CNT textiles demonstrated an increase in the specific electrical and tensile properties (up to 90 and 460%, respectively) without modifying the quantity or quality of the CNTs, as verified by thermogravimetric analysis and Raman spectroscopy, respectively. The enhanced properties were correlated to the degree of CNT alignment within the textile as quantified by small-angle X-ray scattering and scanning electron microscopy image analysis. Clear alignment (orientational order parameter = 0.5) was achieved relative to the pristine material (orientational order parameter = 0.19) at applied field intensities in the range of 0.5–1 kV cm^–1^ at a frequency of 13.56 MHz.

Carbon nanotubes (CNTs) have been extensively studied since the 1990s,^1,2^ and they are now synthesized in myriad forms.^3^ Their physical properties (e.g., tensile strength^4^ and electrical^5,6^ and thermal conductivity^7^) are excellent, especially considering their low density (ca. ≤1.5 g cm^–3^,^8^ and surpass those of most state-of-the-art bulk materials. However, after three decades, CNT technology commercially thrives as additives to plastics, paints, or battery electrodes rather than standalone macroscopic products.^9^ So far, it has failed to replace bulk commodities such as steel, aluminum, or even costly carbon fibers (CFs). The main reason for this shortcoming is the inability to transfer CNTs’ superb nanoscale properties into macroscopic materials.^10^ Post-treatment is a common strategy to improve properties by alignment, doping, or densification. It has been shown that doping with halogens^11^ or acids,^12^ using internal^13^ or external cross-linkers^14^ and thermal annealing,^15^ can enhance properties considerably but with significant limitations such as substantial mass addition (or loss), instability due to dedoping,^16^ or being difficult to scale up.

Another strategy focused on increasing the aspect ratios of CNTs used for wet-spinning, highly aligned fibers.^17^ A power-law relationship was shown relating the fiber’s tensile strength (ratio of 0.9) and electrical conductivity (ratio of 0.8) to the CNTs’ aspect ratios.^18^ Assuming that results from that work can be extrapolated, one may expect well-aligned CNTs having aspect ratios above 10^4^ to outperform any natural or manmade material in terms of strength and conductivity. Thus, work has focused on aligning CNT fibers synthesized by the direct CNT spinning method,^19^ which produces CNTs with aspect ratios of 10^4^ and above.^20,21^ In a recent publication, Lee et al.^22^ mechanically stretched CNT fibers that were immersed in chlorosulfonic acid (CSA), which is considered a true solvent for CNTs.^23^ This allowed the fibers to swell prior to stretching, enabled the debundling of the CNTs, and minimized the chances of mechanical integrity failure that could overshadow any alignment effects. By aligning the CNTs using this method, the fibers’ tenacity surpassed 4 N tex^–1^. These values exceed those of all high-end CFs and are on par with ultrahigh molecular weight polyethylene (UHMWPE) like Dyneema. While this method offers a proof of concept, it is a costly post-treatment involving highly corrosive superacids. More notably, it was less efficient when applied to thicker (>5 tex) CNT fibers, as the CSA had issues infiltrating the bulk of the fiber.^24^ Additional work was done for the in situ alignment of CNTs synthesized in a floating catalyst chemical vapor deposition (FCCVD) reactor. Alemán et al. showed that lowering the concentration of nanotubes in the gas phase reduces the density of entanglements, and the CNT aerogel can be collected at very fast rates (>50 m min^–1^), producing enhanced frictional shear forces that significantly increase CNT alignment and fiber properties.^25^ This work concluded that effective CNT alignment could only be achieved if the alignment mechanism is applied in the gas phase prior the formation and drawing of the CNT aerogel. Although very promising to achieve a higher alignment, diluting the CNT concentration comes with the significant limitation of decreased mass throughput, which affects the cost-effectiveness of the FCCVD process.

An alternative CNT alignment mechanism involves inducing external forces, most notably with electric or magnetic fields, during or after synthesis. CNTs in buckypaper or uncured epoxy composites were aligned by magnetic field intensities of 7–26 T.^26−29^ While promising, these trials were conducted at room temperature. FCCVD reactors work at temperatures above 1100 °C, which is well beyond the Curie temperatures of most ferromagnetic materials. Furthermore, producing such strong magnetic fields requires elaborate and costly setups. The literature also shows various attempts to use electric fields to align dispersed CNTs.^30−32^ The general trend shows that DC fields have minimal effect on alignment, while higher AC frequencies (in the range of MHz) gave better results or required weaker fields (on the order of several kV cm^–1^ rather than tens of kV cm^–1^) to achieve significant alignment.^33,34^ Although encouraging, these trials were performed in liquid media at room temperature for extended periods. To the best of our knowledge, Zhou et al.^35^ have been the only ones to show successful gas-phase CNT alignment by employing an electric field during an ex situ deposition of CNTs for low-density films. They employed a microelectrode array adjacent to the FCCVD reactor’s exit (postgrowth), showing that a significant alignment of ultralong (>50 μm) single-walled carbon nanotubes (SWCNTs) could be achieved for ∼100s of CNTs. It was noted that optimal alignment was attained at an applied field intensity of 2.2 kV cm^–1^ using a 10 MHz AC frequency. It was documented that improved alignment was evident using higher frequencies (in the range from 50 Hz to 10 MHz) and that the DC field showed no alignment. These results align well with our previous theoretical discussion of DC field alignment.^36^

Here we describe the in situ alignment FCCVD system using an AC electric field to enable manipulation and control over the assembly of CNTs in the gas phase for high-density CNT production. Our mesoscale model confirmed that using an AC electric field was necessary to introduce a z-pinch mechanism that stiffened individual ultralong (>300 μm) CNTs, allowing those to align according to the field lines by utilizing field intensities that would avoid gas breakdown. As opposed to previous work, the current process does not involve precursor feed manipulation and does not rely on CNT dilution to enhance the frictional shear forces.^20,25^ Small-angle X-ray scattering (SAXS) and SEM image analysis data reveal a clear trend of CNT alignment with increasing field intensity, which dramatically enhances the electrical and mechanical performance of the end material. This work provides a demonstration and theoretical explanation of continuous in situ CNT alignment via external electric fields and provides a complementary processing technique for enhancing bundle thickness, densification, and CNT alignment to enable higher-performance macroscopic CNT materials.

## Results and Discussion

### Continuous CNT Alignment Using an Internal RF Electrode

**Figure 1:**
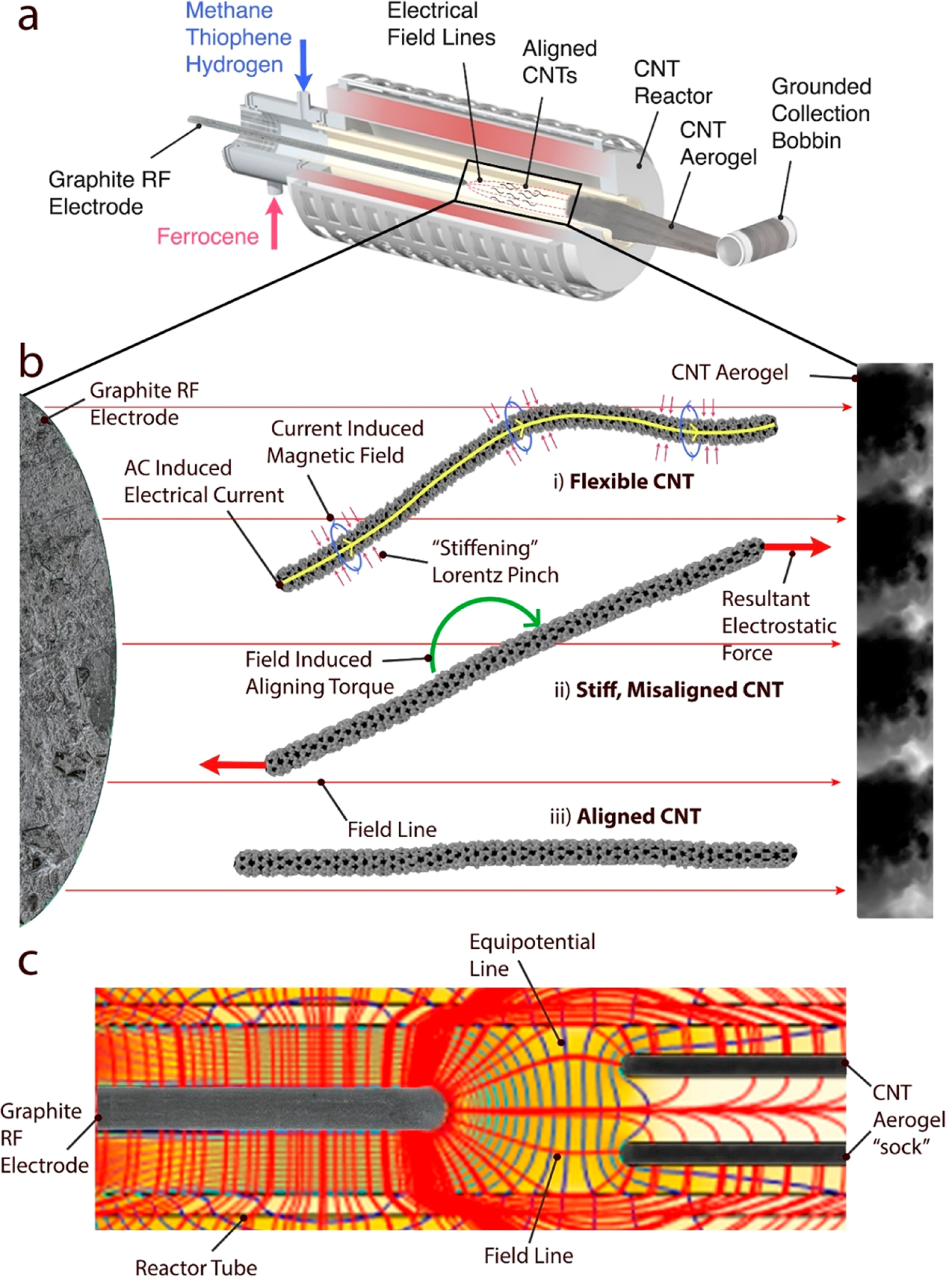
AC field alignment system. (a) An illustration showing the adapted FCCVD reactor with an RF electrode inserted at its front, while the forming CNT aerogel, being collected on a grounded bobbin, acts as a counter electrode (antenna). The CNTs align along the resultant field lines before forming the aerogel. (b) A magnified schematic shows the occurrence in the interelectrode gap. (i) The AC field induces a “Lorentz pinch” that stiffens the ultralong CNT. (ii) The stiff, ultralong CNT is under the influence of a field-induced aligning torque. (iii) The CNT is aligned according to the field lines. The schematic is not to scale, and (i–iii) co-occur. (c) Finite element method numerical results of the field distribution inside the reactor tube portraying equipotential lines (blue) and orthogonal field lines (red). The CNT aerogel (“sock”) was approximated as a 28 mm OD cylinder. The packing density of the equipotential lines indicates the local field intensity. The model shows the presence of alignment-inducing field lines bridging the two electrodes within the interelectrode gap (50 mm wide).

The field alignment adapted FCCVD rig used a single graphite electrode, termed the radio frequency (RF) electrode, connected to the system’s high voltage (HV) unit and inserted through the reactor’s head. The electrically conductive CNT aerogel (continuously synthesized in the reactor) was collected as a nonwoven mat on a conductive bobbin, acting as a grounded electrode (Figure 1a). Once the process ended, the specimens were prepared by rolling the CNT mat out of the bobbin, forming a ring of fiber-like material that was used for further analysis. The aerogel collection speed was low and set to be ∼0.16 m s^–1^, which is a rate that did not induce changes in the CNT network.^20,25^Figure 1b portrays the proposed mechanism of the CNT interelectrode alignment process. As illustrated, we hypothesize that an alignment mechanism results from a generated dipole aligning torque, supported by an AC-induced z-pinch stiffening effect (as discussed further below). Figure 1c illustrates the finite element electric field distribution model of the inner reactor cavity. The model visualizes the axial field, which facilitates the CNT alignment in the interelectrode gap, encompassing the end of the RF electrode and the CNT aerogel “sock” (acting as a receiving antenna) as depicted by the red field lines.

**Figure 2:**
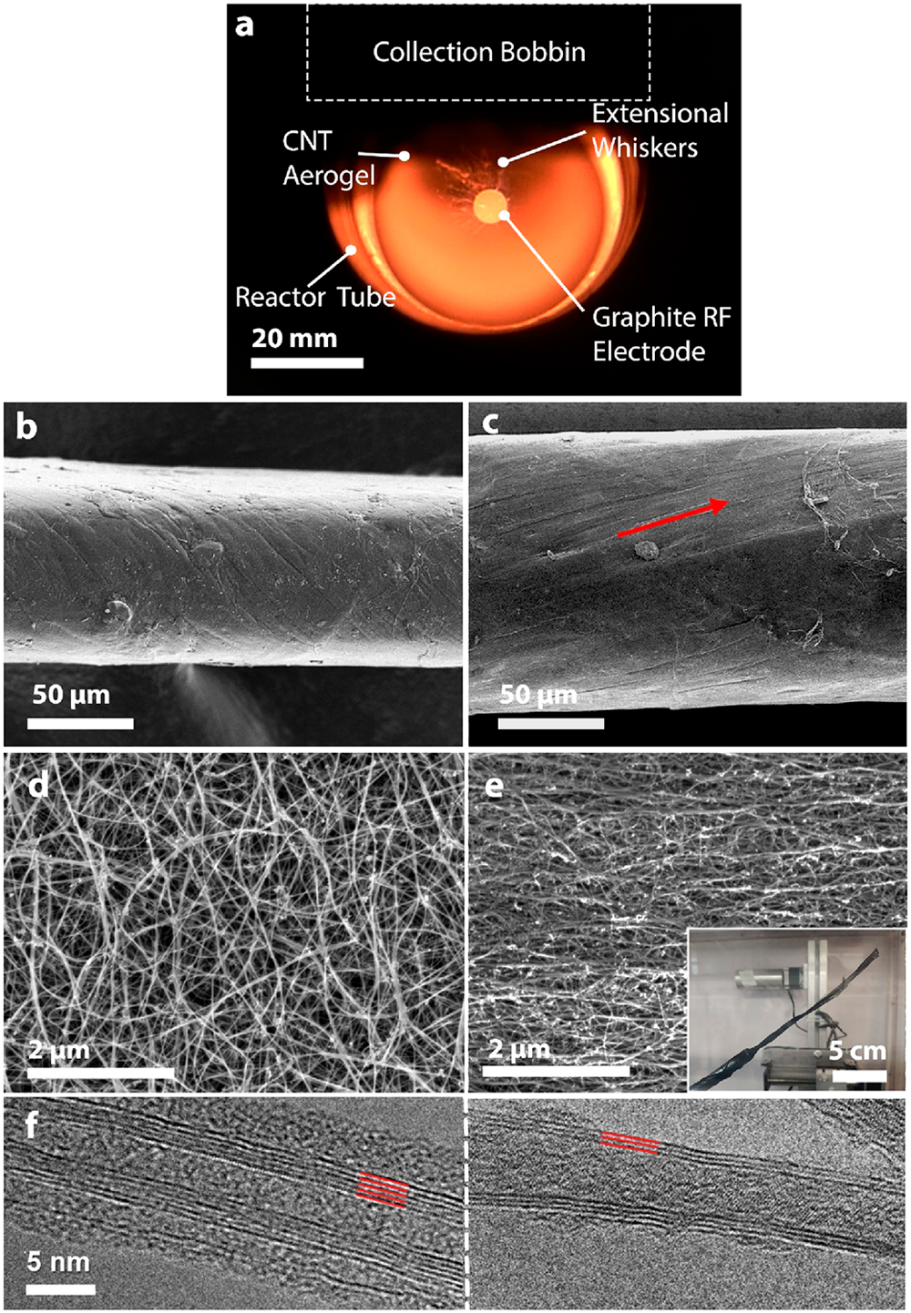
Continuous CNT alignment using an internal RF electrode. (a) An image taken from the reactor’s end directed upstream. The image was taken as CNT aerogel collected on a rotating bobbin when the AC field was applied (250 W). Extensional whiskers are “growing” axially from the end of the graphite RF electrode toward the forming aerogel. (b) Low-magnification SEM image of a cigar-rolled pristine CNT textile sample. (c) Low-magnification SEM image of a cigar-rolled field-aligned CNT textile sample. Even at low magnification, a hint of alignment is evident (red arrow). (d) SEM image showing the isotropic microstructure of the pristine CNT textile. (e) SEM image revealing an aligned CNT pattern within a field-aligned material. (inset) A 15 cm long, single CNT sock produced during AC alignment. The sock is more rigid than absent alignment, being able to support its weight. (f) TEM images of a reference sample show the widespread presence of few walled MWCNTs with three to five walls (red lines).

**Figure 3:**
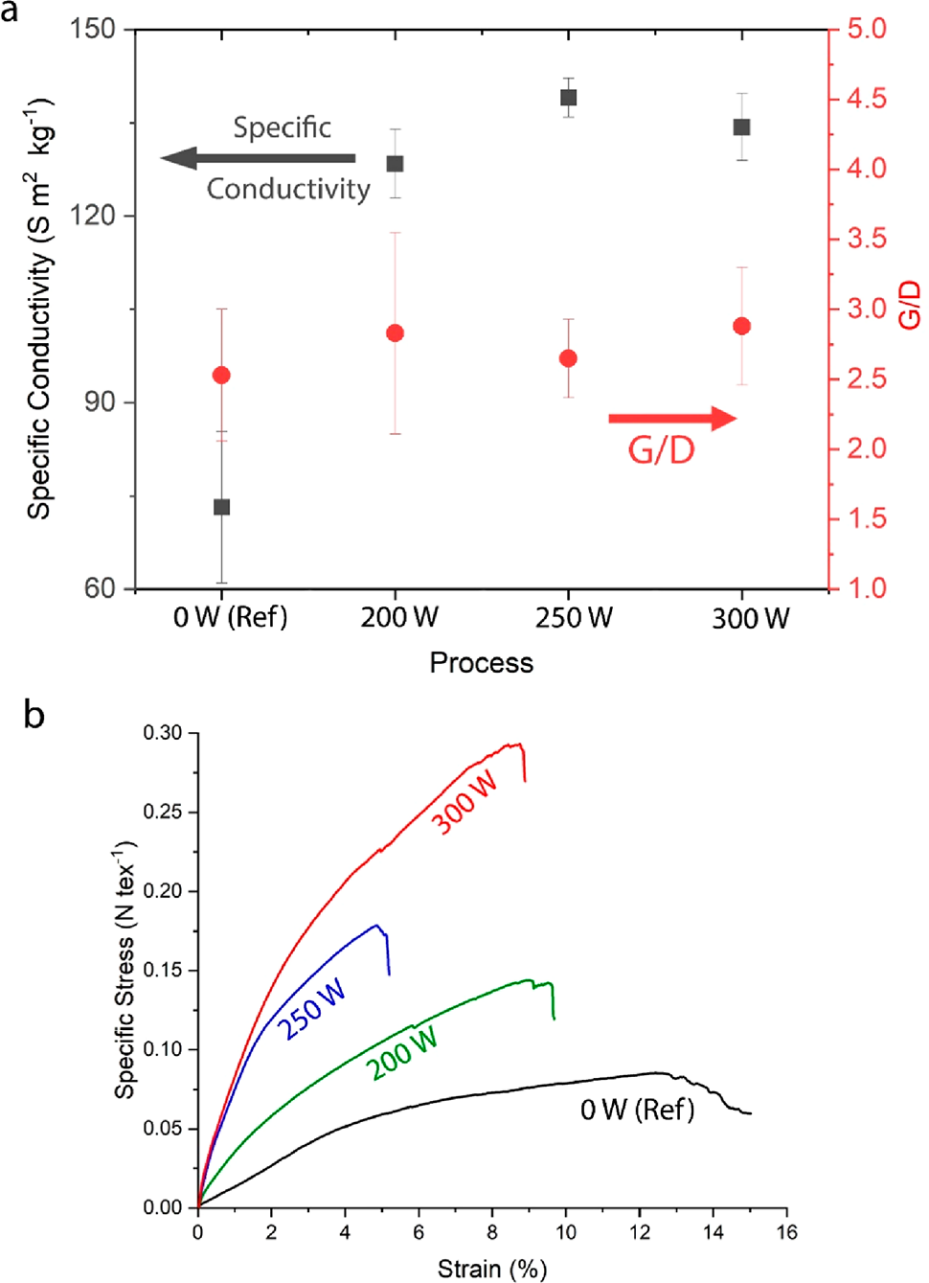
Physical properties of aligned CNT materials. (a) A plot showing specific electrical conductivity (black, left axis) and Raman G/D ratios (red, right axis) of CNT materials collected under different applied AC field intensities and a reference material (0 W). While the G/D ratio did not change significantly, the specific electrical conductivity increased by up to 90%. Error bars denote standard deviation using at least three different samples. (b) Stress/strain curves of tensile measurements show a distinctive change in the mechanical performance from ductile (0 W) to more brittle behavior for aligned samples. The mechanical shift in properties correlates well with the applied field intensity (∝*P*^1/2^).

**Figure 4:**
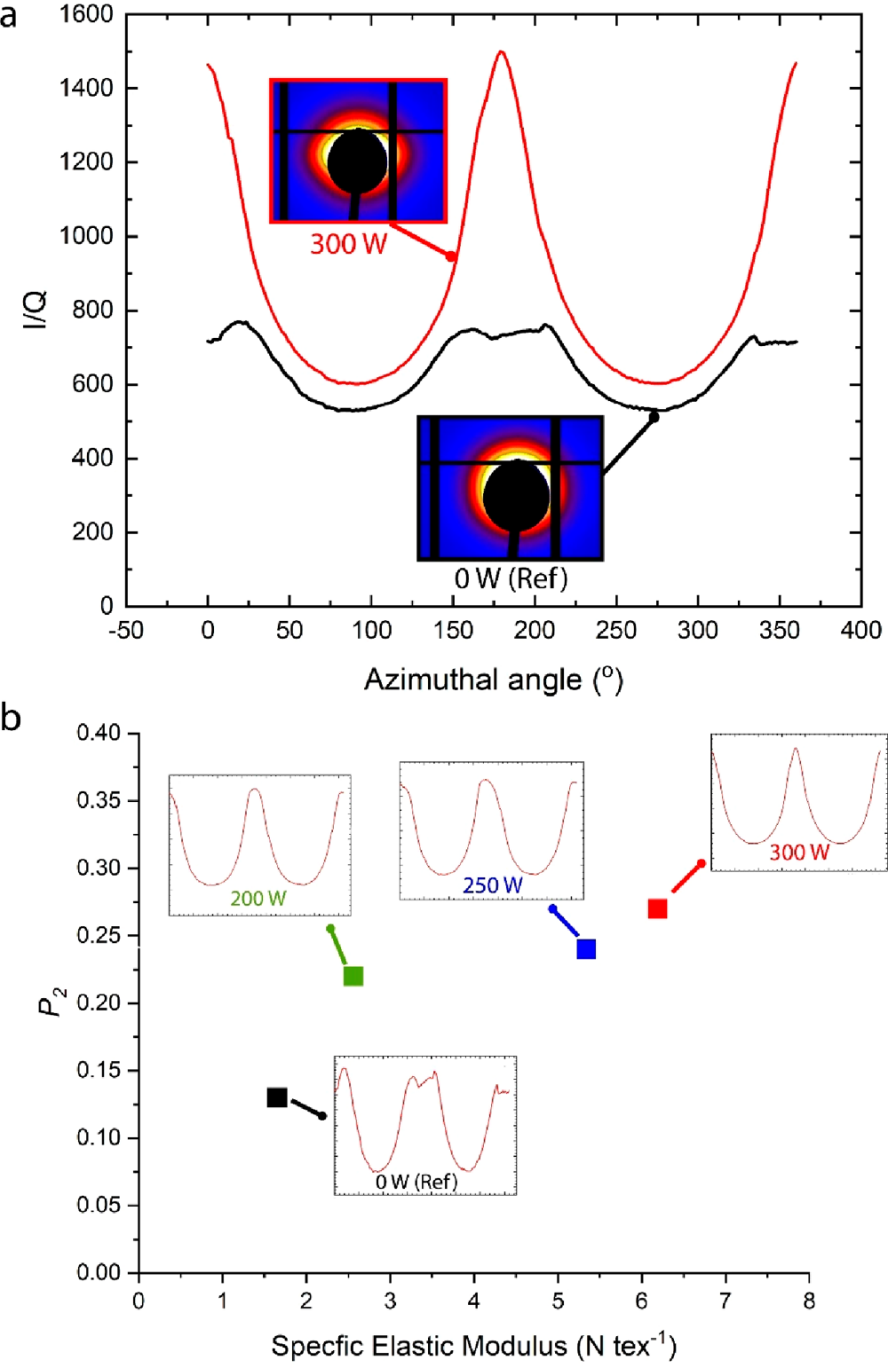
SAXS orientation of CNT materials. (a) Intensity-normalized azimuthal scans of the 0 W (ref) and 300 W samples at Q range from 0.7 to 0.8 nm^–1^. Azimuthal angle, φ = 0°, corresponds to the *x*-axis (equator) and perpendicular to the fiber-like textile axis (insets). Corresponding 2D SAXS patterns. The reference material does not show any apparent scattering pattern, confirming the anisotropic nature of the textile. The 300 W sample shows a distinctive Lorentzian intensity distribution, confirming the presence of CNT alignment. (b) Plot showing the Hermans parameter (*P*_2_) as calculated from the azimuthal scans (insets) vs the sample elastic modulus.

Trials with this single electrode setup were run with the RF power supply set to 0 (reference), 200, 250, and 300 W (maximal system’s power output), and CNT textiles were collected continuously (Figure 2a). Apart from the applied HV, no changes in the process parameters were made, so a direct comparison to the reference runs could be made. Low-magnification scanning electron microscopy (SEM) micrographs show that the produced specimens have a fiber-like morphology with a diameter of ∼100 μm (Figure 2b,c). Some hint of preferred orientation is observed in the material produced under the AC field even at low magnification (Figure 2c, red arrow). Additional SEM imaging revealed that, while the pristine material showed an isotropic microstructure (Figure 2d), a pattern of CNT alignment is easily noticeable in the CNT materials produced under the influence of the AC field (Figure 2e). This material also appeared more rigid, self-supporting its weight without collapsing (Figure 2e inset). TEM imaging confirmed that the current FCCVD process produced mostly multiwalled carbon nanotubes (MWCNTs) with few walls (Figure 2f). Electrical measurements of the various samples showed an increase of 75–90% in specific electrical conductivity, while there was no apparent change in the G/D ratios (based on peak intensities) retrieved from Raman spectroscopy (Figure 3a). Moreover, the Raman profile patterns remained comparable between all the samples (Figure S1). The lack of radial breathing modes (RBMs) validated the lack of SWCNTs in the textile. A thermogravimetric analysis (TGA) has shown that the weight fraction of the CNTs within the textiles is comparable in all the different samples, ranging from 69.6 to 74.6 wt % (Figure S2). These findings indicate that the increase in electrical conductivity was due to microstructure reconfiguration, which led to a lower number of resistive CNT-CNT junctions rather than an improvement in CNT quantity or quality (crystallinity). A mechanical analysis of the modified samples demonstrated a distinctive shift in their tensile behavior, as seen by the stress/strain curves (Figure 3b). The reference CNT material (0 W) showed a ductile behavior with a high (>10%) strain ratio to failure and a vague breakpoint, typical of direct-spun CNT mats.^37^ In comparison, all CNT materials produced under an AC field had a brittle pattern with a lower strain ratio to failure and a clear failure point. SEM imaging of the fracture surface revealed CNT bundle pullouts due to a disentanglement failure mechanism (Figure S3).^37,38^ It also confirmed the shift in the mechanical behavior, from ductile to brittle, as more pullouts are seen, and a pronounced aligned CNT pattern next to the fracture surface is noticed in the samples produced under the use of AC. This shows that, in such samples, more CNTs took part in the load-bearing process from the initiation of the tensile load, leading to higher tenacity yet an abrupt (brittle) failure. These images coincide well with the documented microstructure of failed CNT textiles, either aligned^39^ or isotropic.^40^ On average, a dramatic increase of 260%, 270%, and 320% in the tenacity (specific tensile stress to failure) of the 200, 250, and 300 W samples, respectively, was achieved (Figure S4). This trend is relatively linear considering that the system’s input voltage (and thus applied field intensity) is proportional to the square root of the RF generator power (*V* ∝ *P*^1/2^). Such a striking transformation in mechanical behavior is reassuring evidence for changes in the load-bearing microstructure of the CNT network due to CNT alignment, as documented and discussed in detail in the literature.^20,24,25,37^ To directly evaluate the degree of alignment, an SAXS analysis was performed on the samples. Figure 4a shows the overlaid azimuthal scans of the reference (0 W) and 300 W samples normalized by the invariant (the scattering power) accompanied by the relevant two-dimensional (2D) SAXS patterns. It can be seen that, while the reference sample does not show any orientation pattern (as it is inherently isotropic), the 300 W sample shows the distinctive Lorentzian type of distribution associated with a more profound alignment pattern.^41^ An additional analysis based on raw data integration to calculate the sample’s Hermans parameter (*P*_2_) revealed a trend between the applied power (and voltage) to the degree of alignment (Figure 4b). Also seen is a clear correlation between *P*_2_ and the specific elastic modulus, supporting the concept that the stiffness of the CNT network is dominated by the internal alignment of its CNT bundles.^37^ Intriguingly, apart from the *P*_2_ values, the distribution patterns of the azimuthal scans (as seen in the insets) shifted from Gaussian-like (200, 250 W) to Lorentzian (300 W). As Lorentzian distributions are commonly affiliated by a more profound CNT alignment, this is another indication of how the alignment evolved with the increase in applied field intensity.^41^

To further enhance the applied field intensity in the interelectrode gap, the RF electrode was introduced deeper toward the forming CNT aerogel. However, this resulted in the radial growth of whisker-like materials from the electrode surface outward (Figure S5a). Such a phenomenon is likely to happen due to the intense field surrounding the RF electrode, as represented by the dense stacking of blue equipotential lines seen in Figure 1c. An SEM analysis confirmed that those whiskers were made from networks of submicron vapor-grown carbon fibers (VGCF; Figure S5b,c).^42,43^ It seemed like radial whisker growth hindered a continuous CNT sock collection. However, with the correct positioning of the RF electrode (at 95 mm upstream to the reactor’s midpoint at ∼1100 °C), only axial whisker growth was observed (Figure 2a and movie S1), and continuous sock collection was achieved. In such a manner, the whiskers acted as an extension to the RF electrode (Figure S6) and, thus, narrowed the interelectrode gap, allowing the applied field intensity to be sufficient for achieving noticeable CNT alignment. Although VGCFs grew from the electrodes, none were observed in the gas, nor were any VGCFs incorporated into the textiles.

### CNT Alignment Using an RF Field–A Theoretical Model

#### Z-Pinch Stiffening

To validate the experimental results and clarify the AC field-alignment mechanism, a detailed theoretical model was developed. In general, when an electric field is applied to a CNT, polarization effects will cause the CNT to align with the field. The key difference between DC and AC electric fields is that, in the latter, there is a presence of an axial electrical current within the CNT that constantly changes direction. This contrasts with a simple DC field, where no current will flow after the initial polarization of the CNT. The axial current is remotely induced by the electric field due to the capacitance of the CNT and does not require electrical contact of the CNT to the electrodes of the setup. In the AC case, alignment is still possible, as the polarization of the CNT follows the field direction. While the field magnitude changes sinusoidally, the axis of the field stays the same, and therefore, the polarization leads to an aligning torque toward the field direction. In this work, we show that the axial current is essential in reaching substantial in situ alignment of CNTs using electric fields.

**Figure 5:**
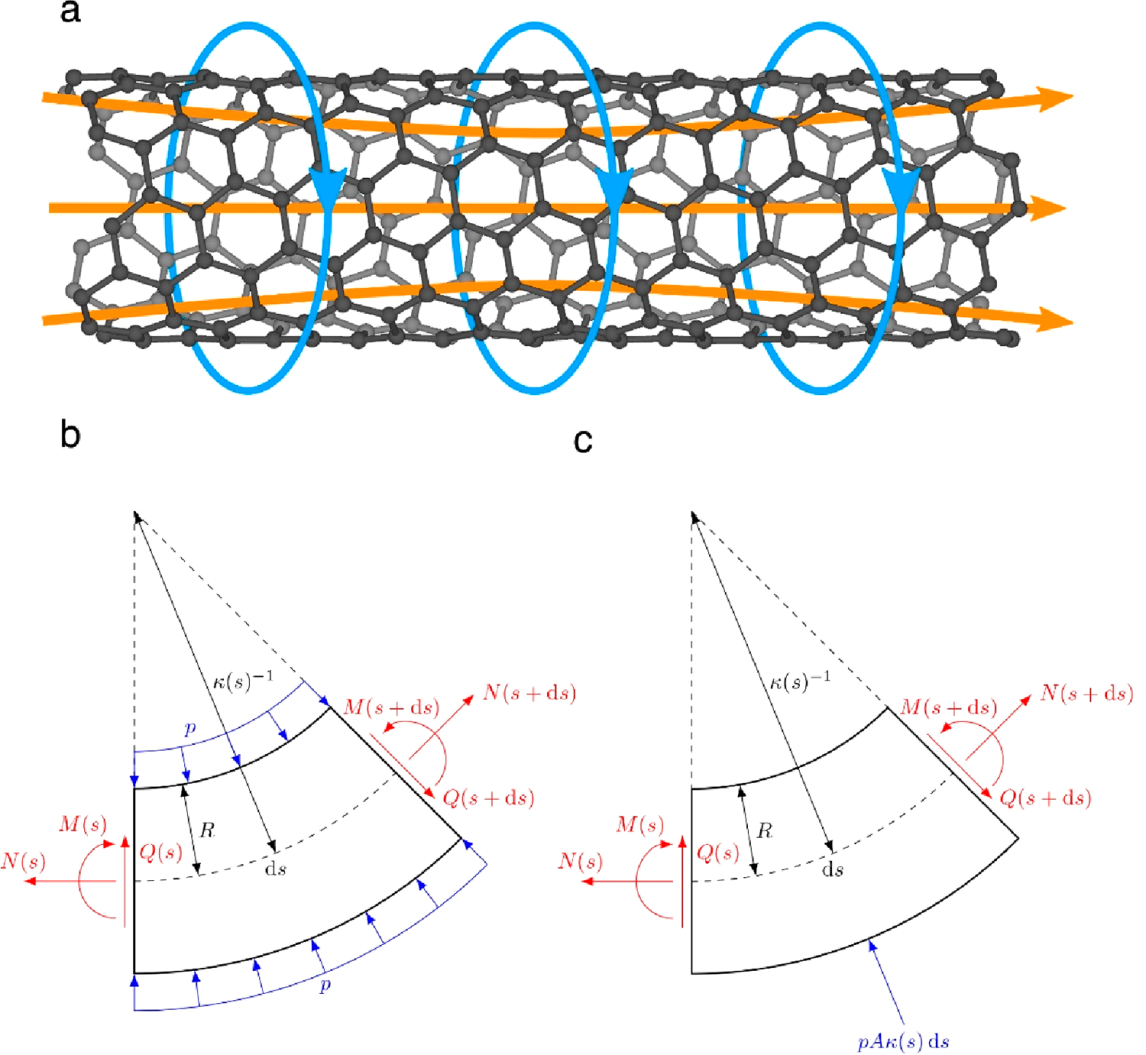
Z-Pinch mechanism. (a) Illustration of electromagnetic fields in a CNT relevant for the z-pinch stiffening effect. Axial current (orange) is confined to the CNT walls and induces a circumferential magnetic field (blue). (b, c) The cross-section free-body diagram of the continuum CNT model for the z-pinch. Internal forces on both faces along the contour are shown in red. Pressure acting on CNT wall (b) and equivalent restoring force (c) are shown in blue.

The axial electric current in the CNT induces a circumferential magnetic field within the CNT wall, as shown in Figure 5a. The axial electric current subsequently experiences a Lorentz force due to the presence of the magnetic field. Effectively, this can be modeled as a pressure acting on the wall of the CNT. We refer to this effect as a “z-pinch”, which refers to this “pinching” of the CNT orthogonal to its longitudinal *z*-axis and is derived from the similar effect used to compress a plasma strongly enough to undergo nuclear fusion.^44^ While the effect is less drastic in a CNT, it can stiffen the CNT to facilitate alignment.

The CNT is modeled as a continuous shell with vanishing thickness. As a mean-field approximation, we assume that the current density within the CNT wall is constant along the entire CNT contour. The current in a CNT is limited by the scattering of the electrons with optical phonons.^45^ Modeling of the current-carrying modes within a SWCNT suggests that electric currents for RF electric fields should exceed the maximum saturation current of a CNT wall of *J*_0_ ≈ 25 μA.^46^ Hence, as we assume current saturation takes place, we will assume that an SWCNT carries the saturation current *J*_0_ when an RF AC field is applied. Furthermore, experiments suggest that, in bundles of SWCNTs and MWCNTs, each CNT wall carries its own saturation current. Hence the total current scales proportionally with the number of walls present in a CNT bundle.^47^ Assuming a constant current density within the CNT shell, the magnitude of the magnetic field can be calculated using Ampère’s law. Subsequently, the Lorentz force density follows directly from the magnetic field and the electrical current density. By taking the limit of vanishing thickness of the CNT shell, an effective Lorentz pressure can be computed.

If a curved CNT segment is considered, the concave side facing away from the center of curvature is compressed, and the convex side is stretched. Hence, there is more surface area for the Lorentz pressure to act on the convex side, leading to an effective restoring force. As this force counteracts any curvature, the CNT is stiffened by the z-pinch effect. An illustration of the pressure and restoring force is shown in Figure 5b,c.

A discussion of the model assumptions and a summary of the intermediate steps of the chain of effects described above are summarized in the methods sections with corresponding formulas, and the individual results are derived in the Supporting Information.

#### Model Results

The primary measure we use to quantify alignment is the two-dimensional orientational order parameter *T*_2_ defined by
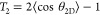
1where Θ_2D_ denotes the two-dimensional alignment angle of the CNT with the electric field. This quantity can be easily measured in two-dimensional SEM images of CNT materials, hence allowing for the direct comparison of our theoretical model with experimental data. The mean value of *T*_2_ varies along the CNT, being lowest at its ends and highest at its midpoint. As a conservative estimate of alignment, we consider the minimum value *T*_2,min_, which is found at the CNT ends.

#### Rigid-Elastic Transition

**Figure 6:**
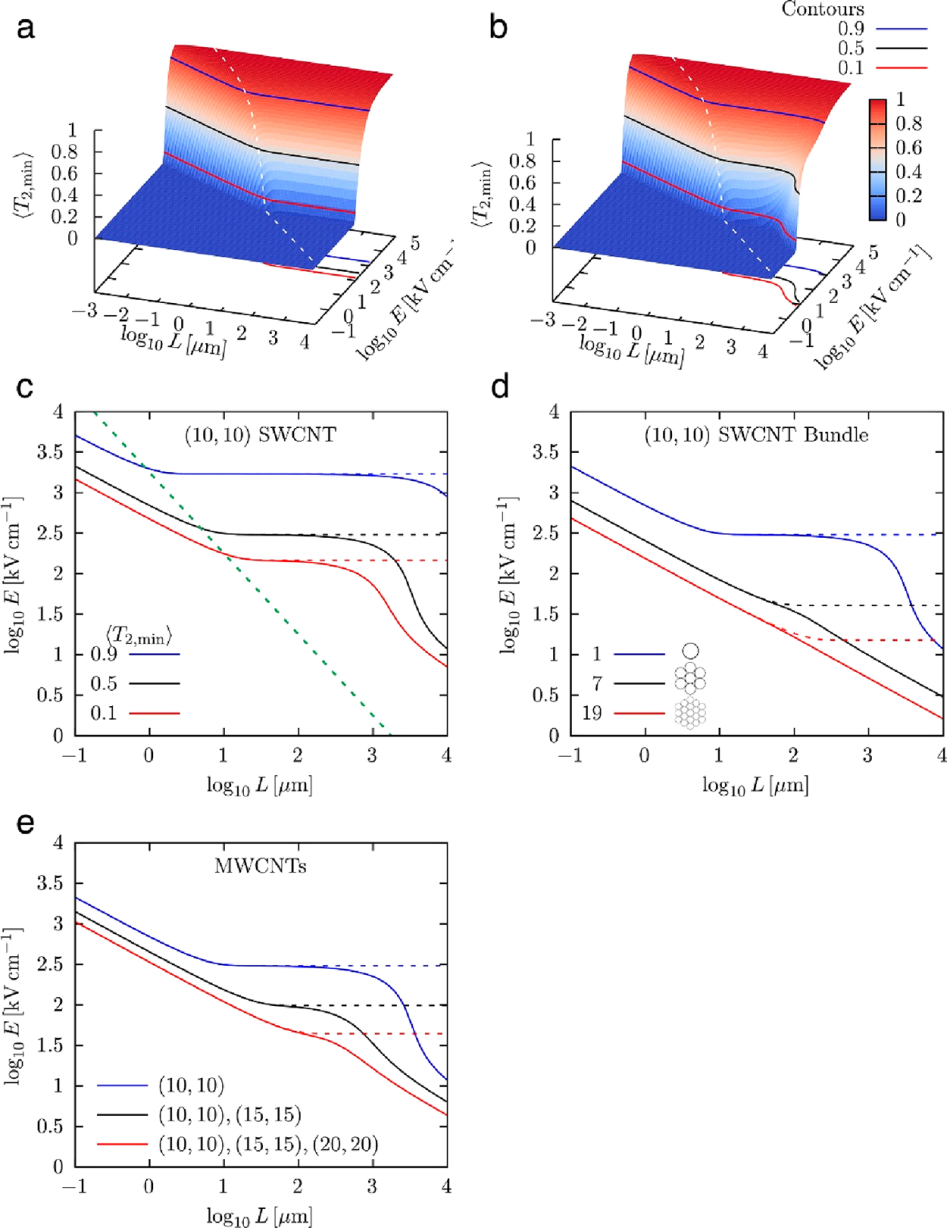
Modeling of CNT electric field alignment. (a, b) Surface plot of *T*_2,min_ (lin) vs CNT length (log) and electric field strength (log) for DC (a) and AC (b) electric fields. Contour lines at different values of *T*_2,min_ are drawn in red, black, and blue. Dashed white line indicates rigid-elastic transition for DC fields. (c) Log–log plot of electric field strength vs CNT length for contours taken from DC fields (a) (dashed) and AC fields (b) (solid). Dashed green line shows the rigid-elastic transition. (d, e) Log–log plot of electric field strength necessary to reach *T*_2,min_ = 0.5 vs CNT length for different (10, 10) SWCNT bundles (d) and MWCNTs with different armchair walls (e).

Intuitively, CNT alignment improves with increasing electric field strength and CNT length up to a certain point. In the DC case, there is a clear change in behavior where *T*_2,min_ no longer depends on the CNT length above a threshold length (Figure 6a). In previous theoretical work, we showed that this threshold length can be derived analytically and is proportional to the persistence length of CNTs.^36^ Below the threshold length, CNTs can be treated as rigid; above the threshold, elastic bending dominates the system, limiting the coupling of the CNT to the electric field. In the AC case, the rigid regime still exists, but for lower values of *T*_2,min_ and long CNTs, the behavior deviates from the elastic regime and returns to the rigid regime (Figure 6b). This indicates that the z-pinch effect stiffens the CNTs, that is, can effectively render them rigid. For SWCNTs that are substantially aligned, this effect only sets in at millimeter length scales (Figure 6c) and is limited by the relatively low value of the saturation current. However, this result demonstrates that z-pinch stiffening can, in principle, facilitate alignment even for SWCNTs.

#### SWCNT Bundles and MWCNTs

The strength of z-pinch stiffening is limited by current saturation in SWCNTs. However, the saturation current scales proportionally to the number of CNT walls in a bundle of SWCNTs or single MWCNTs.^47^ Hence, z-pinch stiffening should be significantly more pronounced in both cases. Figure 6d,e shows the electric field strength *E* that is necessary to reach a certain value of *T*_2,min_ plotted against the CNT length *L* for different bundles of (10,10) SWCNTs and MWCNTs. For the plots, we chose *T*_2,min_ = 0.5 to represent a material with substantial alignment. Both plots contain a single (10,10) SWCNT for reference, where z-pinch stiffening only becomes dominant for millimeter-scale lengths. Once approximately three CNT walls are present, either as individual SWCNTs in a bundle or as a wall of a MWCNT, the most dominant nanostructure in our aerogels (Figure 2f), z-pinch stiffening is already significant at the rigid-elastic transition threshold length. Hence, the z-pinch phenomenon can effectively stiffen CNT structures containing upward of three CNT walls, facilitating their electric field alignment. The electric field strength necessary for alignment then drops below the typical dielectric breakdown field strength of the FCCVD process gas (H_2_ breakdown at 10–20 kV/cm),^48^ making the alignment of single MWCNTs and small-diameter SWCNT bundles technically feasible.

#### Twin-Electrode Configuration

**Figure 7:**
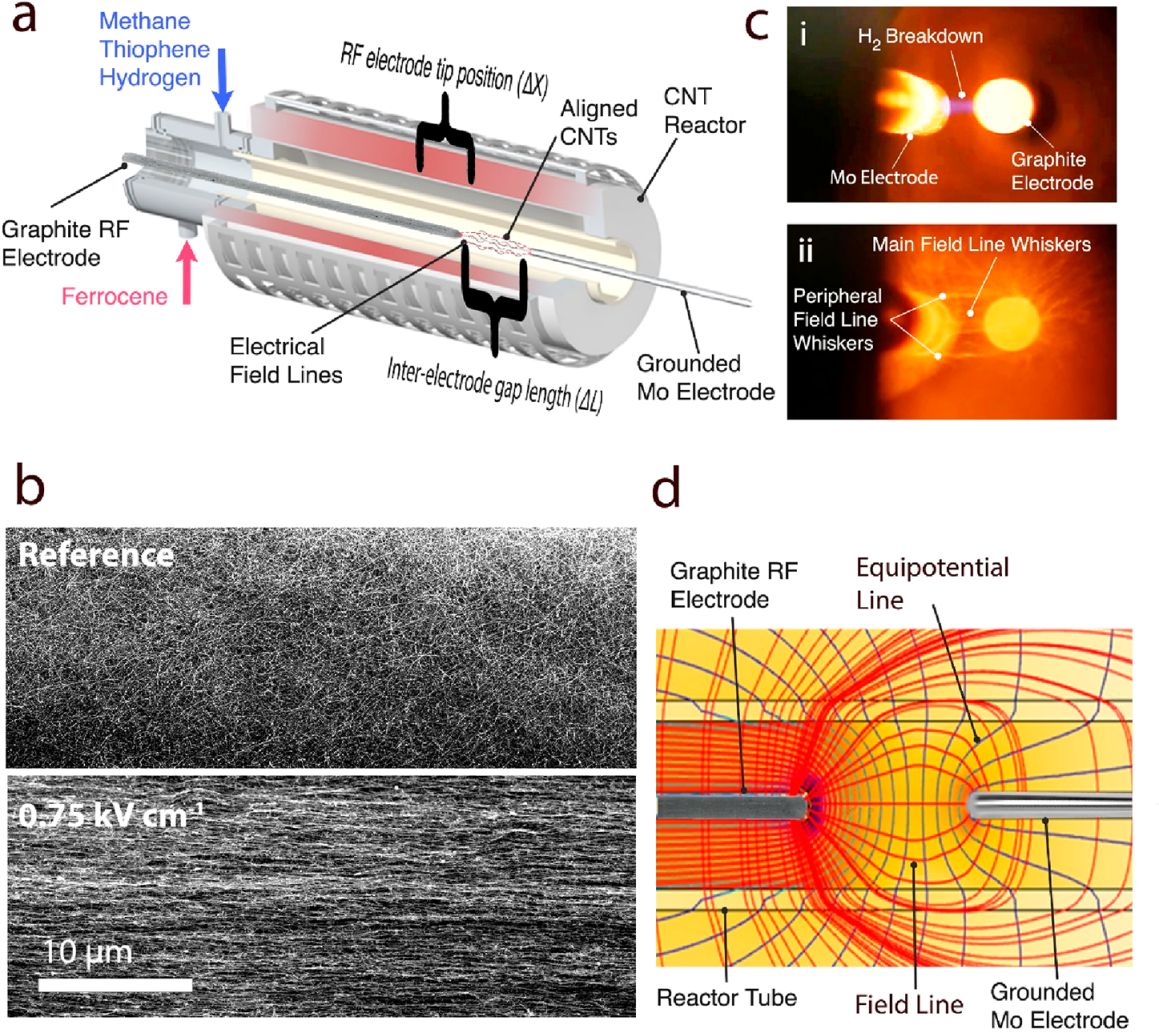
Twin-electrode configuration. (a) An illustration showing the twin-electrode setup with an RF electrode (graphite; 6 mm) inserted at the front and the grounded electrode (Mo; 6 mm) set through the downstream end. Both electrodes can run freely through the central axis, enabling one to control the depth (Δ*X*) and length (Δ*L*) of the interelectrode gap. The CNTs align along the resultant field lines. (b) Low-magnification SEM images showing the isotropic CNT network nature of a reference material (no voltage; top) in comparison to a highly aligned CNT micromorphology seen in a textile produced under the influence of an applied field intensity of ∼0.75 kV cm^–1^ (bottom). (c) A photo of the interelectrode gap. (i) Hydrogen breakdown is witnessed due to the high field intensity (in the range of 10–20 kV cm^–1^). (ii) VGCF whiskers grow in the interelectrode gap according to the bridging field lines between the electrodes. (d) FEM numerical results of the field distribution inside the furnace cavity portraying equipotential lines (blue) and orthogonal field lines (red). The packing density of the equipotential lines indicates the field’s local intensity. The model shows an alignment-inducing field in a 50 mm interelectrode gap, similar to what is manifested in (c,ii).

The single-electrode setup could not allow a proper assessment of the applied field intensity because the interelectrode gap distance was ill-defined as it relied on the aerogel CNT as the grounded electrode. To better define the gap distance and produce greater field intensities, a twin-electrode setup was introduced (Figure 7a). The same graphite RF electrode as in the original setup was employed in conjunction with a second grounded molybdenum counter-electrode, which was inserted through the downstream end. Both electrodes were aligned along the central axis of the reactor’s tube and could freely move longitudinally. This configuration allowed the independent setting of each electrode’s position and interelectrode gap length (Δ*X* and Δ*L*, respectively, as seen in Figure 7a). By specifying the RF power input, better control was achieved over the applied electric field intensity and its longitudinal location.

Using the current setup visually revealed a noticeable enhancement in the degree of orientation. As shown in Figure 7b the micromorphology of a reference sample (top) is isotropic, whereas the material synthesized under the influence of an ∼0.75 kV cm^–1^ in situ electric field (bottom) appears remarkably aligned. As the CNT bundles are well-aligned, it is possible to trace several individual bundles running along with the whole frame, making it discernible that a number of bundles are at least 50 μm long and even more than 100 μm in some cases (Figure S7).

To quantify the degree of alignment as a function of the applied field intensity, an SEM image analysis on the various samples was utilized. An open-access program (Fibre COP) dedicated to quantifying the uniaxial orientational order based on 2D images was used to accommodate such a need.^41^ Because of the 2D nature of the data sets, the software calculated the 2D orientational order parameter *T*_2_ (based on the average of the second moment of the Chebyshev polynomial) rather than the more common three-dimensional (3D) Hermans parameter (*P*_2_), as earlier used in the SAXS analysis.

**Figure 8:**
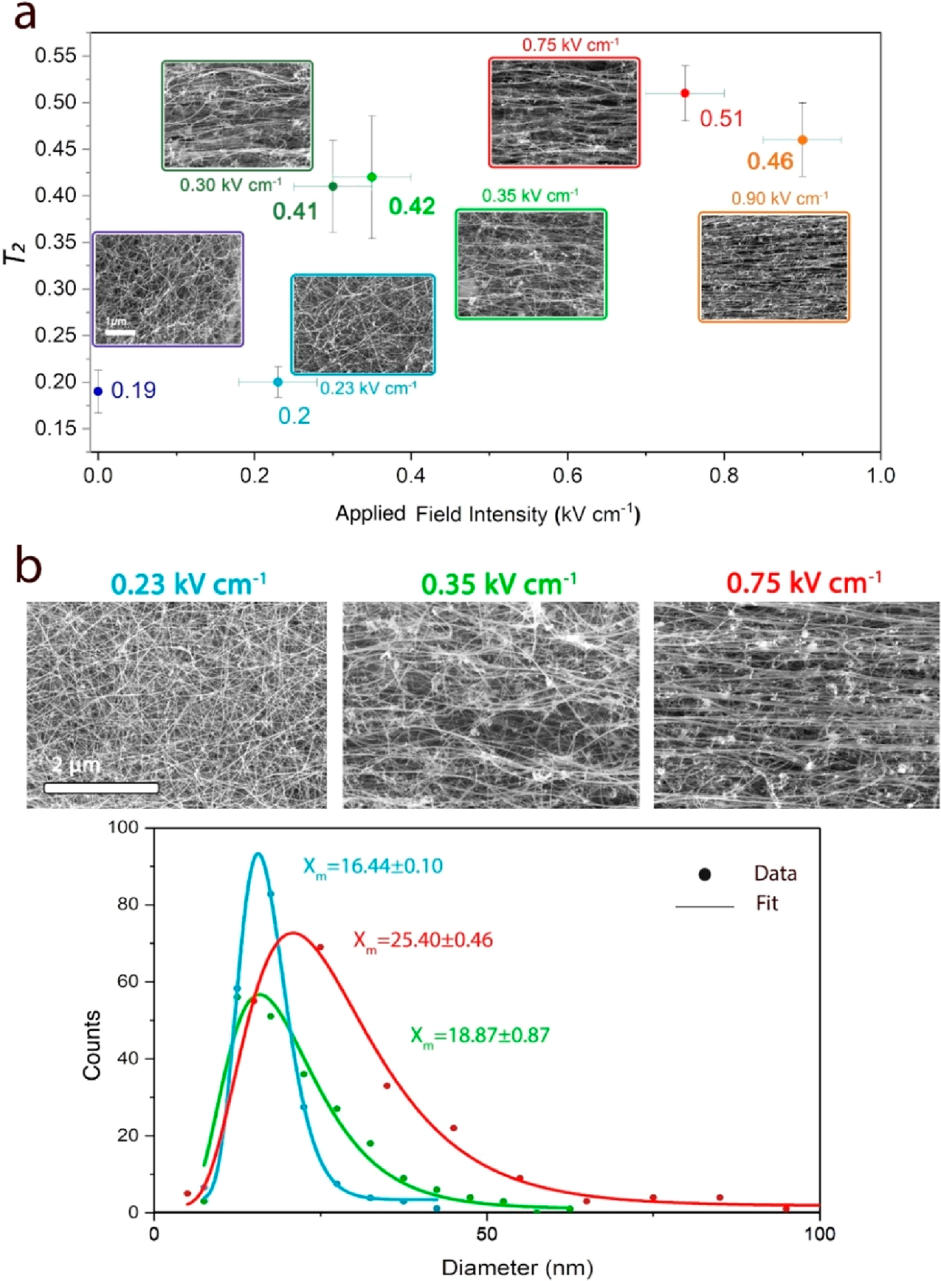
Image analysis of CNT materials. (a) A plot comparing the alignment portrayed by the Chebyshev orientational order parameter (*T*_2_) calculated by the Fibre COP software (accompanied by a typical SEM image) to the applied field intensity generated in the interelectrode gap. While field intensities of less than 0.23 kV cm^–1^ did not seem to affect the alignment, reaching field intensities of more than 0.3 kV cm^–1^ showed a considerable increase in alignment. *Y*-value variance is based on the standard deviation of calculated *T*_2_ values derived from at least three images of two different samples; *X*-value variance is based on the voltage generated in two of the system’s extreme set points. (b) Bundle diameter distribution (log-normal fitting) shows that the median bundle thickness transforms from 16.44 ± 0.10 to 18.87 ± 0.87 and 25.40 ± 0.46 nm for a material produced at a field intensity of 0.23, 0.35, and 0.75 kV cm^–1^, respectively. For each sample, 200 bundle diameters were manually measured.

As indicated in Figure 8a, the reference sample (0 kV cm^–1^) is visually isotropic, exhibiting a *T*_2_ of 0.19 (slight orientation), which can be related to a small degree of alignment in the material due to the associated gas flow in the reactor. Setting the system with an applied field intensity of 0.23 kV cm^–1^ seems not to change the fundamental isotropic nature of the CNT aerogel, leaving the orientation parameter without an actual change at 0.20. Only when the field intensity was enhanced to 0.30–0.35 kV cm^–1^ was a noticeable CNT alignment pattern revealed. While a portion of the CNT bundles did not follow the horizontal pattern, a vast fraction did and, as a result, increased the *T*_2_ value to 0.41–0.42. When the field intensity was increased to 0.75–0.95 kV cm^–1^, a very distinctive alignment pattern became noticeable. An image analysis revealed that the orientation parameter rose to 0.46–0.51, which was found to be very similar to values calculated from SEM images taken from commercial densified CNT fibers (TorStran 5 tex, Tortech Nano-Fibers Ltd.). This increase in *T*_2_ from ∼0.19 to ∼0.5 is significant, as the nonlinear relation equates to a reduction from ∼100° to ∼43.5° in the full width at half-maximum (fwhm).^41^

In addition, as there is evidence in the literature that waviness can influence the mechanical properties of CNT textiles,^49^ we further quantified the waviness of the CNTs by measuring the curl ratio of CNT bundles in the SEM images. The curl ratio is defined as the ratio of the contour length divided by the end-to-end distance of a bundle.^50^ It was shown that the curl ratio is generally close to its minimum value of 1.0, with 75% of traced bundles having a curl ratio of less than 1.075 at all applied field intensities, including the reference sample without any applied electric field (Figure S8). This indicates that the CNTs are primarily straight in all considered samples, which may be explained by the high stiffness of bundles made up from ultralong CNTs. Furthermore, there is a weak trend of decreasing mean and median curl ratio with increasing field, but no such trend can be observed for the curl ratio variation. The decrease in curl ratio and hence waviness may be attributed to the alignment of the CNTs with the electric field as well as the stiffening of the CNTs due to the z-pinch effect. As the curl ratio does not differ substantially from its minimum value in all cases, we primarily attribute any property improvements to an increase in alignment.

The system could be tuned to create a gas breakdown between the two electrodes, a phenomenon that requires field intensities of 10–20 kV cm^–1^ for a hydrogen atmosphere and confirms the system can reach HV (Figure 7c,i and movie S2).^48^ A visual manifestation of the field distribution was evident when whiskers grew between the electrodes (Figure 7c,ii and movie S3). The geometry of these whiskers corresponded well with the field line patterns represented in red by the updated field distribution model (Figure 7d). The local field intensity in the vicinity of the CNTs is enhanced by the high aspect ratio of one-dimensional (1D) nanostructures.^51^ As a first-order approximation, such an enhancement factor is proportional to the aspect ratio (∼10^4^) of the 1D nanomaterial and, according to a more precise approximation, should be ∼500.^52^ This should explain why the experimental applied field intensity was efficient even if it was an order of magnitude lower than the value predicted by the mesoscale model to reach a *T*_2_ of ∼0.5 (Figure 6d).

Interestingly, after running substantial experimental setups with various Δ*X* and Δ*L* configurations, it was evident that CNT alignment is only achieved if the grounded electrode is positioned at least 140 mm downstream from the furnace’s midpoint. This result coincides well with our previous understanding that most of the CNT aerogel synthesis occurs at the last third part of the reactor.^53^

Another change observed in the material’s micromorphology was associated with the CNT bundle diameter. As shown in Figure 8b, the higher the field intensity employed in the interelectrode gap, the thicker the CNT bundles became. The CNT median diameters were analyzed to be 16.4, 18.9, and 25.4 nm for field intensities of 0.23, 0.35, and 0.75 kV cm^–1^, respectively. Our recent study on collision rates between CNTs^54^ and the alignment of CNTs into bundles^55^ has shown few parameters with which to vary the number of CNTs per bundle and, thus, alter the fundamental aerogel structure. We hypothesize, on the basis of our previous work, that the characteristic collision time for aligned tubes increases (i.e., longer time between collisions) as the collision cross-section of two parallel linear structures is smaller. Additionally, the characteristic reorientation time for tubes upon collision will decrease, as aligned tubes will be significantly oriented in the same direction. Thus, the increase in characteristic collision time and decrease in alignment time scales will serve to increase bundle diameters and may delay the onset of gelation. Therefore, the presence of inter-CNT electrical and Lorentzian pinch forces on the CNTs due to the AC field enables one to modify the basic bundle structure of macroscopic CNT materials, resulting in the thicker bundles shown in Figure 8b.

## Conclusions

We have developed an approach that utilizes external electrical fields (up to an intensity of ∼1 kV cm^–1^) to form a substantial effect on the self-assembly mechanism of CNTs in the gas phase, as manifested by apparent CNT bundle thickening from ∼16 to ∼25 nm. The primary innovation of the system is the enabling of continuous in situ manipulation of the nanomaterials while these are being collected to form macroscopic textiles. As determined by SAXS, the method has proven to generate distinctive alignment patterns compared to the isotropic nature of the original bulk material. This microstructure reorganization nicely correlates with the textile’s mechanical behavior transition from ductile to brittle, increasing the elastic modulus by up to 459%. As the alignment led to a higher portion of load-bearing nanotubes resisting tensile load, the specific stress to failure increased by up to 422%. This also led to fewer resistive CNT-CNT junctions with an associated electrical enhancement of up to 90%. Interestingly, the electric field did not influence the CNT synthesis process, as no apparent CNT crystallinity changes could be detected using Raman spectroscopy, and the CNT fraction within the textile remained unchanged as clarified by TGA. A well-developed mesoscale model validated the experimental results by showing that AC-induced electric currents stiffen CNTs and enable alignment at lower field strengths, an effect that is absent for DC fields.

We show a successful attempt to utilize electrical fields for the in situ manipulation and control of the assembly process of CNT networks in the gas phase. The single RF internal electrode design allowed the continuous collection of the CNT textile even though it prevented the utilization of maximal field intensities. Although not designed to continuously produce CNT textiles, the twin-electrode setup enabled us to fix the electrode spacing during aerogel production, thus allowing us to determine the alignment efficiency versus the applied field intensity. These insights from both setups motivate future work encompassing new reactor system design utilizing external electrodes, enabling continuous CNT collection at higher field intensities.

CNT textiles have already shown great potential in various applications such as water^56^ and air^57^ filtration, oil–water separation,^58^ and ballistic protection^40^ as well as lightweight current collectors of electric double-layer capacitors^59^ and lithium ion batteries.^60,61^ Nonetheless, optimization of the alignment process via external AC electric fields can dramatically enhance the properties of the high aspect ratio (∼10^4^) CNT-based textiles without sacrificing the efficiency and cost-effectiveness of the FCCVD process. Enhancing the tenacity of CNT textiles to values on par with standard carbon fibers (CFs; ∼2 N tex^–1^) would enable the adoption of CNT fibers in place of CF. CFs are commonly adopted to lower weight and thus energy consumption of automotive and aerospace vehicles. However, CF production requires several time-consuming (>100 m long plant),^62^ consecutive heating steps (up to 3000 °C in the carbonizing stage) and, as such, has associated embedded emissions of 20–36 kg of CO_2_ per kilogram of produced CF.^63,64^ Conversely, a direct-spun CNT textile requires a considerably shorter single production step (∼5 m reactor), processed at a temperature of ∼1300 °C, to have the potential for production at a fraction of the environmental impact and production cost. In addition, CNT textiles are dramatically more flexible than CFs,^39^ leading to a mechano-structural advantage, and as they are more electrically conductive, they can be applied more efficiently for intrinsic self-sensing concrete.^65^ A broader adoption of CNT textiles to replace high carbon intensity materials, such as steel (1–2 kgCO_2_/kg) and aluminum (5–7 kgCO_2_/kg),^66^ will only occur through densification and alignment. Thus, processes that achieve alignment will be used if they achieve efficient, cost-effective, and scalable production of aligned and densified CNT textiles.

Our approach can also prove attractive for further manipulations on the process, such as affecting the precursor cracking or catalyst growth dynamics, and complements many postprocessing densification and functionalization processes that have already been developed. The alignment of CNTs and the densification of CNT materials has been highlighted as a means to enhance properties. Recent studies have shown that postprocessing techniques, such as superacid stretching,^22,67^ can serve to dramatically enhance the properties of CNT fibers originating from CNT aerogels. The underlying structure of the CNT network influences the final properties of the resulting postprocessed materials when the bundles are not fully dispersed. Therefore, CNT AC field alignment enables a higher degree of orientation at the outset of in situ condensation (e.g., acetone capillary condensation) or ex situ stretching processes and thereby serves to complement existing techniques for bulk fiber processing. Through the combined means of in situ CNT process control and ex situ enhancements, CNT orientation and densification are likely to continue the effective doubling of strength and conductivity properties every three years, as has been demonstrated over the past decade.^68^

## Methods

### High-Voltage System

A cabinet was fabricated to act as an RF-shielded compartment for the high voltage (HV) components, ensuring personnel and equipment safety. This housed a 300 W RF generator (Dressler Cesar 1312) working in the license-free 13.56 MHz band. The generator’s output was connected to a 50 ohm load through a series-connected L-C circuit tuned to 13.56 MHz. The arrangement resulted in a high power being generated at the connection between the inductor and the capacitor. A second variable capacitor was connected in parallel with the inductor, so its effective reactance could be varied. The L-C junction was connected to an RF electrode inside the reactor. The voltage was tuned by modifying the reactance of the series capacitor and the parallel combination of the inductor and its capacitor, according to the equation below
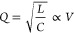
2where *Q* is known as the voltage magnification factor, *L* is the effective inductance, *C* is the capacitance, and *V* is the output voltage.

The RF output voltage was measured by connecting a resistive voltage divider (985 kΩ + 1 kΩ) across the high-voltage output of the network and measuring the voltage across the 1 kΩ resistor using an oscilloscope (72–8705A Tenma) and a 1:1 probe, with 30 W applied input power. A correction was applied to account for the stated input impedance of the probe. For given settings of the capacitors, the output voltage is proportional to the square of the applied power, so the measurements at 30 W have been appropriately scaled.

### Finite Element Modeling

The field distribution inside the furnace was modeled using the AC/DC module of COMSOL Multiphysics. The small dimensions of the furnace’s interior (overall length 500 mm) compared with the free-space wavelength (22 m) allowed us to model the field on a quasi-DC basis. In such a model, the form of the electric field is independent of the applied voltage. The CNT aerogel seen in Figure 1c was modeled as a cylinder with an outside diameter of 28 mm and an inside diameter of 25 mm.

### Continuous CNT Alignment by a Single RF Electrode

The FCCVD reactor was equipped with a 6 mm graphite electrode (Beijing Great Wall Co.), referred to in the text as the RF electrode. The electrode was connected to the HV system and was inserted into the reactor through a bespoke injector flange. This flange enabled the free lateral movement of the RF electrode, while side ports were used to introduce the process precursors. The electrically conductive CNT aerogel forming within the reactor was collected on a conductive bobbin, grounded by a copper strap. This arrangement created an axial electric field between the RF electrode and the grounded aerogel. The RF electrode tip was stationary and positioned 95 mm upstream from the reactor’s midpoint at a temperature of ∼1100 °C. The reactor was operated with the RF generator output power set in turn to 0, 200, 250, and 300 W. Reflected power during each collection was minimal (<10 W). Each power configuration run was repeated at least three times. After each collection ended, the CNT material was manually rolled perpendicular to the collection axis to produce a “cigar-rolled” thin string on the bobbin’s circumference. The string was cut at a random point to produce an ∼160 mm long CNT linear thread. The linear density of the produced CNT textiles was between 7 and 15 tex (=g km^–1^). In all FCCVD runs, unless specified otherwise, the process ran as follows: the furnace was set to 1300 °C, precursors included hydrogen (1400 standard cubic centimeters per minute; sccm, BOC); methane (160 sccm, BOC); ferrocene (200 sccm of hydrogen through a tank heated to 110 °C, 98% purity Merck); thiophene (60 sccm of hydrogen through an ice-slush cooled reservoir at ∼0 °C, ≥99% purity Merck). Collection speed was set at 30 rpm (a linear speed of ∼0.16 m s^–1^).

### CNT Alignment by a Twin-Electrode Setup

The FCCVD reactor was equipped with two electrodes aligned along the central axis of the 50 mm (OD) alumina work tube (Almath Crucibles; Figure 7a). As in the former setup, a 6 mm graphite electrode was used. An additional 6 mm molybdenum electrode (Goodfellow), referred to in the text as the grounded electrode, was inserted from the far end of the reactor. To ease its alignment and fix the grounded electrode’s position, a grounded *z*-axis translation stage (Optics Focus Instruments Co.) was used. To maximize the electric field homogeneity, both electrode tips were polished to produce hemispherical smooth ends. The experiments were run by discretely varying the interelectrode gap (Δ*L*) from 200, 150, and 130 down to 50 mm. This was facilitated by changing the RF electrode tip position while the grounded electrode end was stationary (140 mm downstream to the reactor’s midpoint). The power supply of the HV unit was set to 300 W (highest output) except for Δ*L* = 50 mm, in which a 0 W (reference) and a 180 W power setting was also used. Each setup was run at least twice. All FCCVD runs employed the same process parameters as described in the continuous CNT alignment by a single RF electrode section.

### CNT Textile Characterization

Textiles were weighed using a microbalance (Sartorius SE2-F), and their length was measured to calculate the linear density of each sample (in g km^–1^), also known as tex.

Textile linear resistance was determined by measuring the resistance of a 100 mm section of each sample using a bespoke four-point probe jig connected to a milliohm meter (Aim-TTi BS407). Specific electrical conductivity was calculated by normalizing the linear conductance (inversely proportional to the linear resistance) according to the linear density of each sample. Specific electrical conductivity units used were S m^2^ kg^–1^. Specific electrical conductivity values were averaged according to a set of at least three samples.

Textile tenacity (ultimate tensile stress normalized by linear density) and strain at failure were determined using an Instron mechanical tester (5500R) equipped with a 10 N load cell. The initial gauge length was 20 mm, and the sample displacement rate was 1 mm min^–1^. Sample pretension was fixed at 0.1 N. To prevent slippage, the ends of the CNT fiber samples were sandwiched and glued between aluminum foils before being clamped to the grips. Fiber tenacity and strain at failure values were averaged according to a set of at least three samples.

Raman analysis was conducted using a Horiba XploRA PLUS confocal microscope system, using a 638 nm laser, 50× objective, 1200 grating, 25% laser power, and three accumulations of 30 s. G/D ratios were calculated based on peak intensities and were averaged according to a set of at least three repeats on three different samples.

TGA analysis was done using a thermogravimetric analyzer (TGA/DSC1, Mettler Toledo). All samples reached equilibrium at 45 °C and then were heated to 900 °C at a rate of 5 °C min^–1^ under a flow of dried air set to 50 sccm.

2D SAXS patterns of CNT materials were collected at ALBA synchrotron light facility at BL11-NCD-SWEET noncrystalline beamline, equipped with Dectris (Pilatus 1M) photon counting and Rayonix LX255-HS CDD detectors. Scattering of the samples was collected using a microfocus spot of ∼10 μm in diameter and at a radiation wavelength of λ = 1.0 Å. Before the patterns were collected, the position of the sample holder was calibrated using silver behenate (AgBh). The collected patterns were first corrected for background scattering and then analyzed using DAWN software (v. 2.20), obtaining azimuthal profiles after radial integration over *Q* range of 0.7–0.8 nm^–1^. The intensities were normalized by the scattering invariant *Q* obtained from Kratky plots, *q*2•*I*(*q*) versus *q*. The *P*_2_ values were computed using a Legendre series approach and assuming full equatorial reflection, providing a lower bound for the values.^69^

For high-resolution transmission electron microscopy (HRTEM) imaging, specimens were prepared by sonicating ∼10 mg of CNT material in 200 mL of 1-methyl-2-pyrrolidinone (NMP, 99% purity; Merck) for 60 min in an ultrasonicator (Hielscher, UP400ST). One milliliter of the dispersion was pipetted on a Lacey Formvar/Carbon TEM grid (Ted Pella) and was left undisturbed for 1 min to be then blotted away. The residual NMP was dried by baking the grid in a vacuum oven at 70 °C overnight. Imaging was done in a high-resolution mode using a monochromated FEI Titan 80–300 TEM operated at 300 kV.

### SEM Imaging and Image Analysis

SEM imaging was performed using an MIRA3 field-emission gun SEM (Tescan). Imaging was done at an acceleration voltage of 5 kV using the In-Beam SE detector at a 3–5 mm working distance. The specimens were not sputter-coated. For alignment quantification, images were acquired at a 50 000× magnification using a 4096 × 3072 raster. In case alignment was visually evident, images were manually taken at an angle that most CNTs were parallel to the long axis of the rectangular frame. At these imaging parameters, the resolution was calculated to be ∼3.8 pixels per CNT bundle (based on the finding that the CNT bundles’ median diameter was between 16 and 26 nm as shown in the results section), and as such, the number of CNTs per frame should be higher than 500. The resolution and number of CNTs per frame satisfied what was required for a successful image analysis, as published by Brandley et al.^70^ An SEM image analysis was performed to acquire the image’s orientational distribution function (ODF) and further extract the orientational order parameter (namely, the second moment, which is the average of the Chebyshev polynomial *T*_2_). The analysis was done by the use of the open-access Fibre COP software.^41^ The program parameters were set for a number of five scans, bin size of 0.25, with a filter interval of five. The number of peaks was set to three, while each peak was Lorentzian-fitted. Acquiring the average *T*_2_ orientation parameter for each twin-electrode experimental setup was based on the analysis of at least three SEM images (a total of more than 1500 CNTs). SEM images for CNT bundle diameter analysis were taken using the same configuration as described above but with a 200 000× magnification. 200 CNT bundle diameters were manually measured using Fiji, and the histogram was fitted by a log-normal distribution using OriginPro 2021.

For the waviness analysis, CNTs were manually traced using the FiberApp software for fitting worm-like chains to the SEM images.^71^ Default fiber tracking parameters were used. For each investigated field intensity, a 2 μm × 2 μm section of the SEM was randomly selected, and at least 50 CNT bundles were traced per image (see Figure S9). The contour length *L* and end-to-end distance Δ*R* of each traced bundle were computed. Using both, we quantified waviness using the so-called curl ratio τ = *L*/Δ*R*.^50,72^

### Modeling of CNT Alignment Under the Influence of an RF Field

#### Model Assumptions

The CNT alignment with alternating electric fields can be described using the worm-like chain (WLC) model with energy contributions from bending,^73^ electric polarization,^36^ and the additional electromagnetic interactions due to the z-pinch stiffening effect introduced in this work. We note that the model only considers individual CNTs or CNT bundles and does not take interactions with other CNTs or any other substance in the CNT material into account. Thus, the model may be understood to describe CNTs right after the formation process, which are not fully incorporated into the network structure of the bulk material yet, or where the bulk material is sparse, and interactions can be neglected. Nevertheless, we expect our model to illustrate the effect of z-pinch stiffening and its relevance in facilitating the alignment of CNTs with AC electric fields. In addition, the model has the benefit of being exactly solvable without the need for mean-field approximations of the alignment field, albeit with substantial algebraic complexity.

Interactions between CNTs can be taken into account by extending the WLC free energy derived in this work by a director mean-field coupling of each CNT with the bulk of the material. Such an approach is often used in models of elastic liquid crystals and is known as the Maier–Saupe theory.^74,75^ Such an approach would also allow one to consider CNT-CNT junction density and residual catalyst content in the coupling strength. Generally, on the one hand, we would expect an explicit inclusion of CNT-CNT interactions (other than within the considered CNT bundles) to further facilitate the alignment of CNTs due to favorable van der Waals attraction. On the other hand, we expect residual catalyst content to be negligible, as the statistics of the alignment behavior of CNTs are dominated by the coupling to the electric field and the elastic properties of the CNTs, neither of which should be significantly affected by the catalyst.

Calculating the coupling strength with the bulk material is a nontrivial task and typically depends on many parameters such as the composition of the material, CNT aspect ratio, and the density of the material.^76^ Thus, an in-depth treatment of either of the interaction effects mentioned above requires careful consideration and is beyond the scope of this work.

Finally, we assume that the current in the CNT is constant in time, along the contour, and equal to the saturation current for each CNT wall. To justify this assumption, we used an existing model for the RF behavior of CNTs found in the literature^46^ and adjusted it for freely suspended CNTs in an AC field (Figure S10). The corresponding calculations can be found in the Supporting Information. By calculating the current distribution in the CNT as a function of time, we show that the time dependence of the current can be neglected in the megahertz regime, as it is below the resonance frequency for CNTs (Figure S11a). Furthermore, we calculate that, for SWCNTs with lengths greater than 300 μm, one can assume that the current saturates nearly along the entire contour of the CNT (Figures S11b,c). Motivated by these calculations, and to make the model mathematically tractable, we assume that the current magnitude is constant in the CNT in both time and along the contour.

#### Current, Pressure, and Force

In the following, we briefly summarize the key results in deriving the free energy expression of the model. The full derivation is provided in the Supporting Information.

As justified above, we start by postulating a constant current *J* in the CNT. In the derivation of the Lorentz pressure, we start by assuming a continuous CNT with a finite wall thickness. This assumption later disappears when taking the limit of vanishing wall thickness but simplifies the calculation of the Lorentz pressure. Using Ampère’s law, it is possible to compute the magnetic field strength inside the CNT wall. The axial electric current and circumferential magnetic field are shown in Figure 5a. The magnetic field and the current in the CNT interact, leading to a uniform compressive Lorentz force on the CNT wall. By integrating over the width of the CNT and taking the limit of vanishing wall thickness, one can show that the Lorentz pressure acting on the CNT wall is equal to
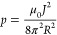
3with *R* being the CNT radius. By further integrating over the surface at each point along the contour, parametrized by *s*, the following restoring line force density can be derived
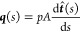
4where *A* = *πR*(2) is the cross-sectional area of the CNT, and **t̂**(*s*) is the tangent vector along the CNT. Hence, the pressure resulting from the current will always work against the curvature of the chain. The pressure and restoring force are illustrated for a 2D continuum model of a CNT in Figure 5b,c.

#### Energy Contributions

Using variational methods, we may further compute the energy contribution of the restoring force density due to z-pinch stiffening.
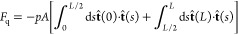
5

This energy has the natural interpretation of both halves of the chain being pulled in the direction of their respective closest ends with the midpoint of the chain being fixed in place.

The current and the pressure that results from it need to be externally induced in the CNT. This can be done by applying an electric field **E** across the CNT. Assuming a simple model where charges can only move tangentially within the CNT, the following energy contribution of the electric field itself has been proposed in some of our previous work^36^
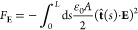
6where *A* is again the cross-sectional area of the CNT.

Combining the energy terms discussed above with the standard curvature term of the WLC yields the full free energy functional of our model.
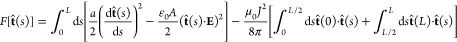
7where *a* simply denotes the bending stiffness of the CNT.

### Harmonic Approximation

For our purposes, it will be sufficient to assume that the CNT is already strongly aligned with the electric field. Without loss of generality, we let the electric field point along the *z*-axis and have magnitude *E*. As is commonly done in the literature,^77,78^ we may then expand the tangent vector and its derivative to second order in the *x* and *y* components of the tangent vector **Θ** (*s*) We then arrive at the following harmonic approximation for the free energy, up to an additive constant.
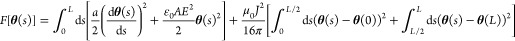
8

This approximate model is the basis for our results and can be solved exactly using methods from Gaussian statistical field theory.^77^ For mathematical details, the reader is referred to our previous theoretical work in which the procedure to solve the model is identical. Details about the parametrization of the model are provided in the Supporting Information. Furthermore, as the model is inherently three-dimensional, we need to calculate *T*_2_ in terms of a three-dimensional average. A corresponding derivation is also found in the Supporting Information.

## Supporting Information Available

Additional sample characterization by Raman spectroscopy (full spectra), TGA analysis, SEM imaging of CNT textiles and their fracture surface, and waviness analysis. Summary of the mechanical properties of the aligned CNT textiles. Optical and SEM images of vapor-grown carbon fibers (VGCF). An in-depth description of the modeling of CNT alignment under the influence of an RF field and how *T*_2_ was derived from a 3D model (PDF)
Video showing the axial VGCF whisker growth during the initialization of HV while running the FCCVD process using a monoelectrode configuration (MP4)
Video showing H_2_ gas breakdown in the interelectrode gap using a twin-electrode configuration (MP4)
Video showing the buildup of a VGCF network in the interelectrode gap during the initialization of HV. Such a network acts as a visual manifestation of the electric field distribution within the gap (MP4)
