# Supplementary material for: Highly Oriented Direct-Spun Carbon Nanotube Textiles Aligned by In Situ Radio-Frequency Fields
Source: ACS Nano. 2022 May 31;16(6):9583–97. doi: 10.1021/acsnano.2c02875 (PMC9245349; doi:10.1021/acsnano.2c02875)
Supplement: Supplementary file 1 — nn2c02875_si_001.pdf [file nn2c02875_si_001.pdf]

Supplementary Information

Highly Oriented Direct-Spun Carbon Nanotube Textiles Aligned by In Situ Radio-Frequency Fields

Liron Issman[1], Philipp A. Kloza[2], Jeronimo Terrones Portas[2], Brian Collins[3], Afshin Pendashteh[4], Martin Pick[5], Juan J. Vilatela[3],James A. Elliott[2] and Adam Boies[1]*

1 Department of Engineering, University of Cambridge, Cambridge CB2 1PZ, United Kingdom

2 Department of Materials Science & Metallurgy, University of Cambridge, Cambridge, CB3 0FS, United Kingdom

3 BSC Associates Ltd, 2 Pilgrims Way, Ely, Cambridgeshire, CB6 3DL, UK

4 IMDEA Materials Institute, c/Eric Kandel 2, Getafe 28906, Madrid, Spain

5 Q-Flo Limited, Buckhurst House, 42/44 Buckhurst Avenue, Sevenoaks, TN13 1LZ, United Kingdom

** e-mail: amb233@cam.ac.uk*

Raman Analysis

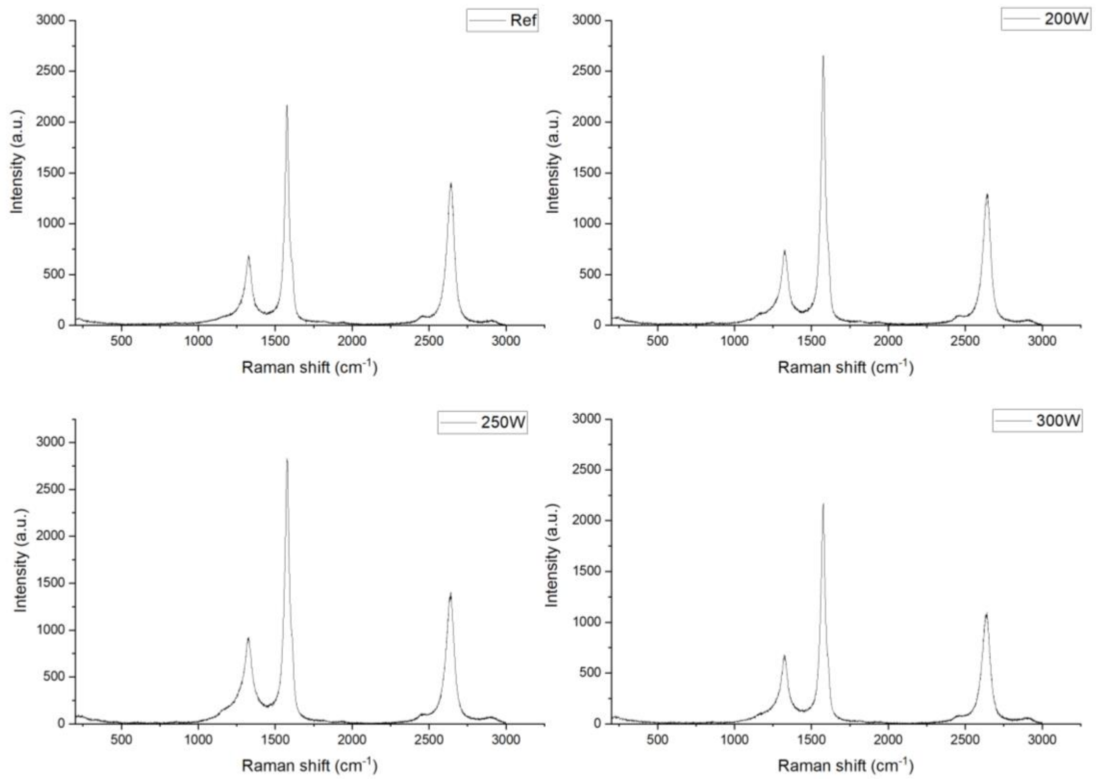

Figure S1. Raman spectra of various CNT samples produced by the internal RF electrode setup. There is no significant difference between the reference sample spectrum to the other spectra of materials produced under the influence of an electric field.

TGA Analysis

TGA analysis revealed that the CNT fraction within the textiles was consistent between all samples, ranging from 69.6 %wt. to 74.6 %wt. (Fig. S2). However, the contents of amorphous carbon (i.e., soot, defective carbon etc.) seemed to somewhat increase with the rise in HV (from ~4 to ~12% wt. in the reference and 300 W samples, respectively). As the G/D ratio of the samples remained similar (Fig. S1), this increase should not be attributed to the change in the CNT quality but most probably to the incorporation of VGCF whisker pyrolysis products (Fig. S5). The greater the HV that was applied, the higher the temperature got in the extensional whiskers (Fig. S6), thus leading to a higher pyrolysis rate in the reducing atmosphere of the reactor. The TGA residue is mainly made from oxidized iron nanocatalysts. Lower residue concentrations in the higher field intensity samples may be a result of electrostatic deposition. This could be attributed to increased losses of floating catalysts due to electrostatic interactions with the reactor walls. These parts consist of the reactor's ceramic tube walls and other grounded metallic parts positioned next to the path of the continuously collected CNT aerogel and the aerosolized nanoparticles surrounding it. The higher the applied intensity used in the process, the more dominant these electrostatic interactions would become, presuming that particles are charged or have induced dipoles, contributing to higher electrostatic losses and lower residue concentrations.

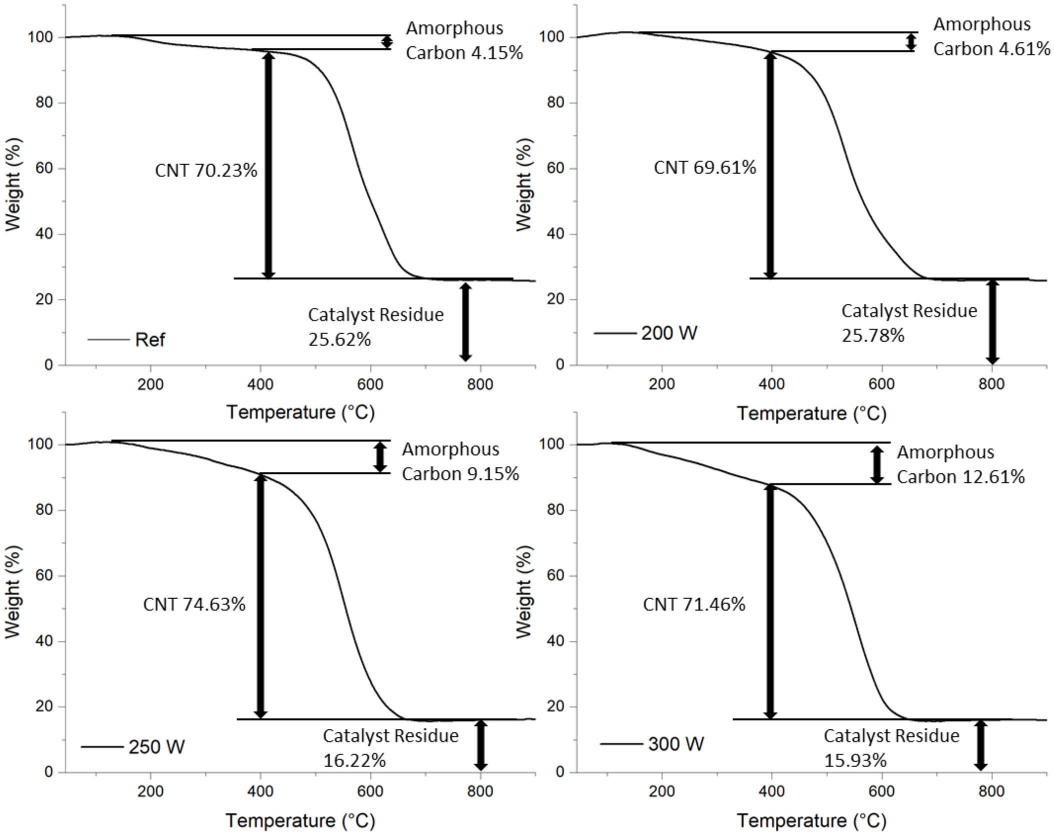

Figure S2. TGA curves of CNT materials produced by the internal RF electrode setup. All runs were in air starting from 45 to 900°C. (a) Reference sample – HV off. (b) 200 W sample. (c) 250 W sample. (d) 300 W sample. CNT weight fraction is similar in all samples.

SEM Fracture Face Imaging

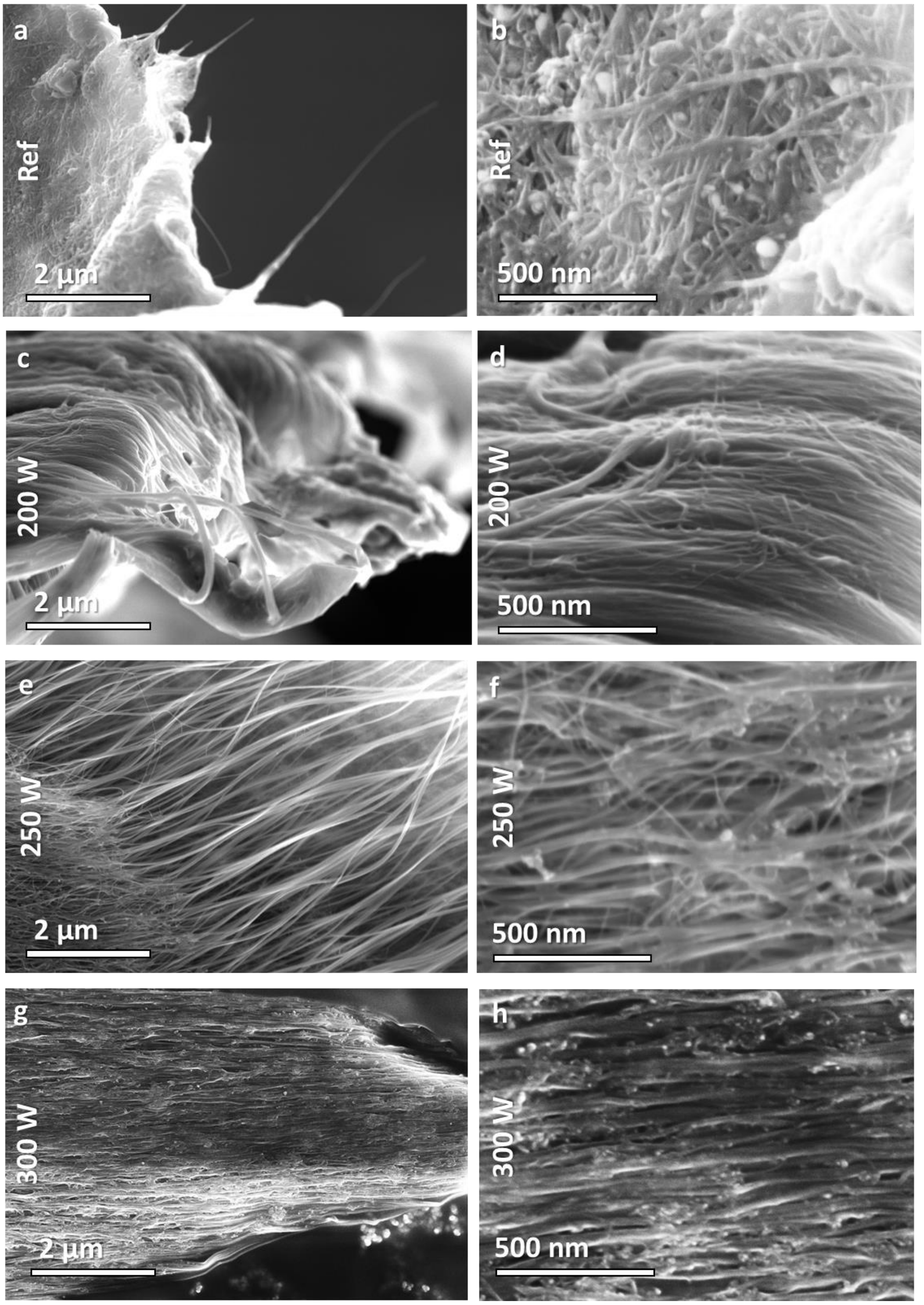

Figure S3. SEM micrographs of the CNT textile fracture surface of materials produced by the internal RF electrode setup. Left column is for low-magnification images, right column is for high-magnification images of the CNTs adjacent to the fracture interface. (a-b) Reference sample (c-d) 200 W (e-f) 250 W (g-h) 300 W. While all fracture surfaces contain CNT pullouts, the HV samples seem to have considerably more. The CNTs next to the fracture surface in HV samples show a considerable amount of alignment, whereas anisotropic nature is present in the reference.

Mechanical Properties Summary of AC Aligned CNT Textiles

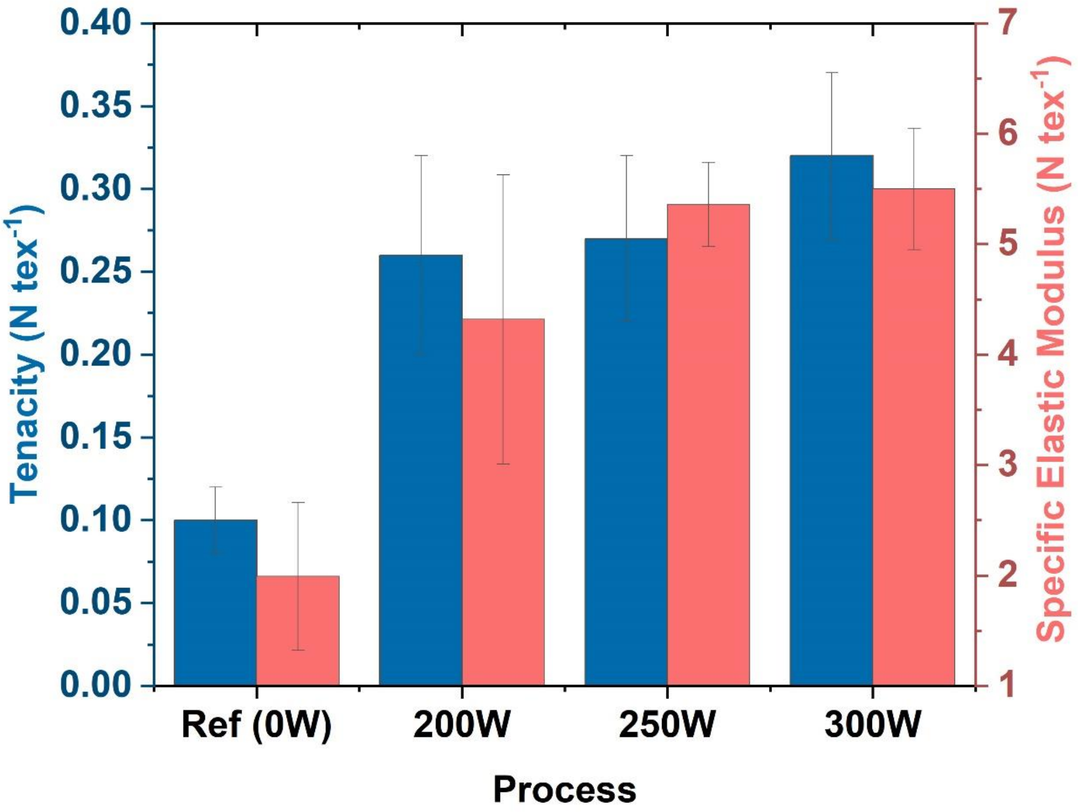

Figure S4. Bar plot summarizing the tensile properties of AC field-aligned CNT samples. The tenacity is shown in blue (referring to the left y-axis), and the specific elastic modulus is shown in red (referring to the right y-axis). Samples included materials produced under an AC field with an RF input power of 200, 250, and 300W and a reference sample collected without any applied field (0W). Error bars denote standard deviations using at least three different samples.

VGCF Whisker Growth in the FCCVD Reactor

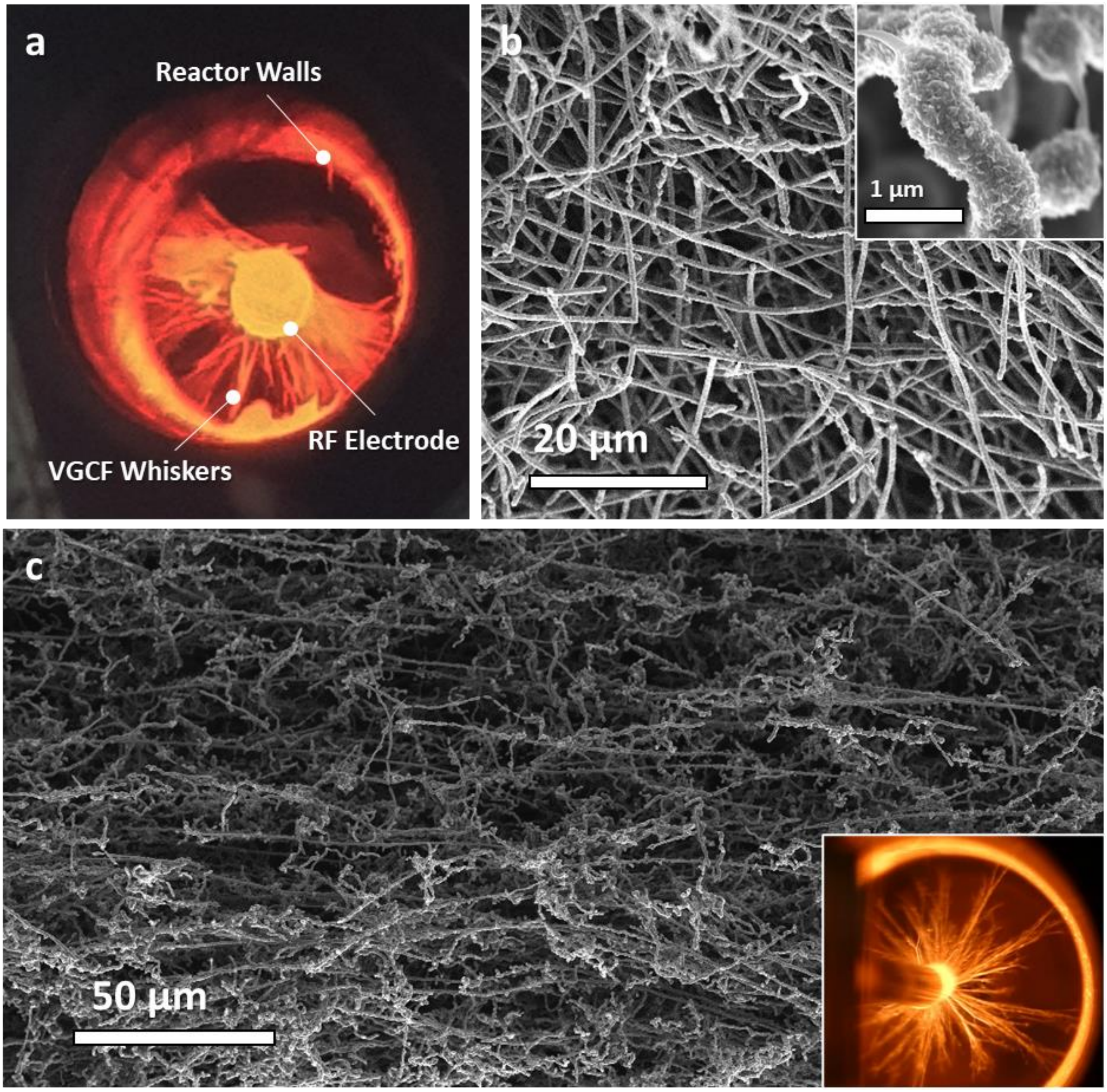

Figure S5. VGCF formation in the FCCVD reactor. (a) VGCF whiskers radially grow from the RF electrode surface towards the reactor's walls. (b) SEM image of a whisker, revealing an isotropic network of VGCFs. Inset shows a single VGCF in higher magnification. (C) SEM image of a whisker produced under the influence of the HV, showing more alignment in the VGCF network. Inset shows that finer "dendrite"- like whiskers are produced when the HV is applied during the whisker synthesis.

VGCF Whisker as Extensional Electrodes

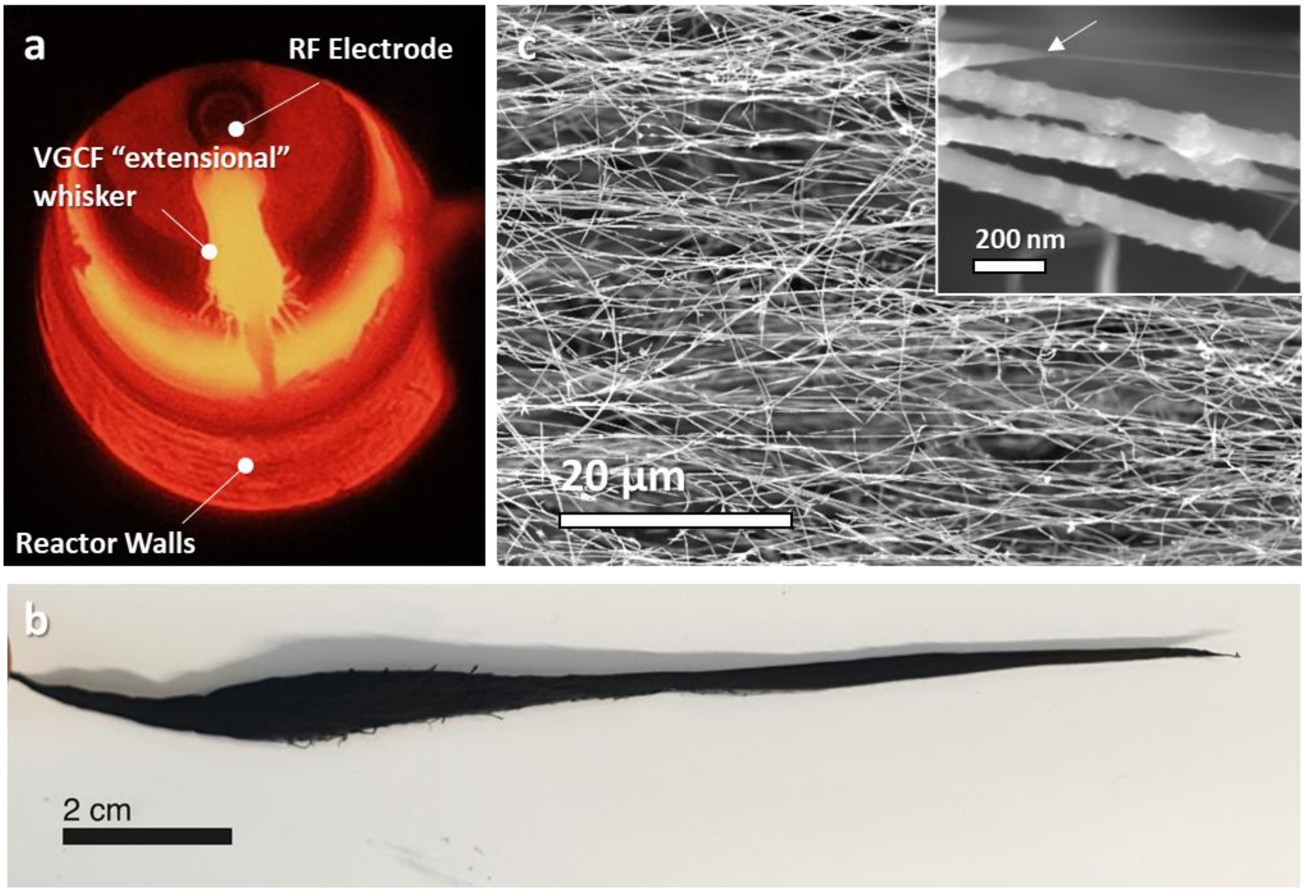

Figure S6. VGCF "extensional" whisker growth in the FCCVD reactor. (a) VGCF whiskers axially grow from the RF electrode downstream, creating an extension to the RF electrode. (b) Some of the "extensional" whiskers grew to be 150 mm long. (c) SEM image of a VGCF "extensional" whisker made of long, aligned VGCFs. The inset shows a high magnification image revealing that the VGCFs are extremely thin (~100 nm in diameter) with a CNT core (arrow).

SEM Imaging of HV Aligned CNT textiles

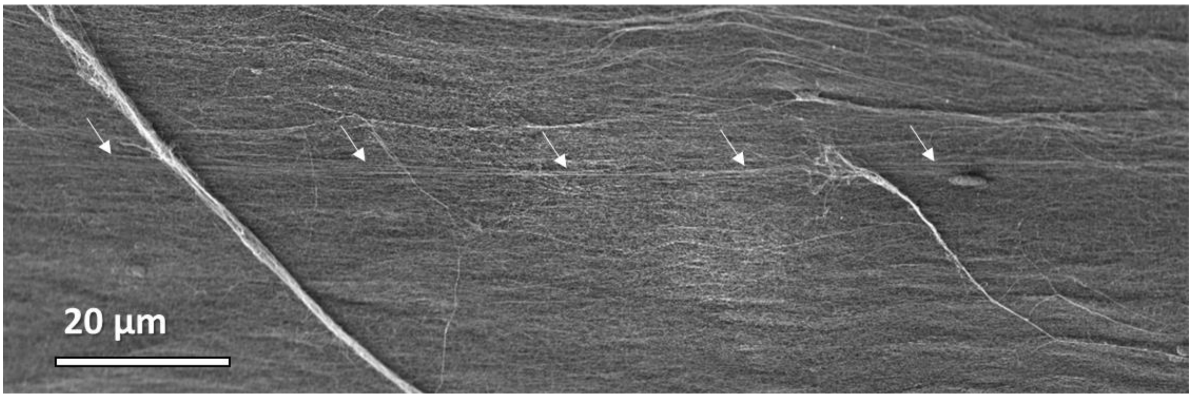

Figure S7. SEM image of CNT material produced under a field intensity of 0.75 kV cm^{-1}. Arrows track the path of an ultra-long CNT bundle measuring more than 100 µm in length.

Summary of Waviness Analysis results

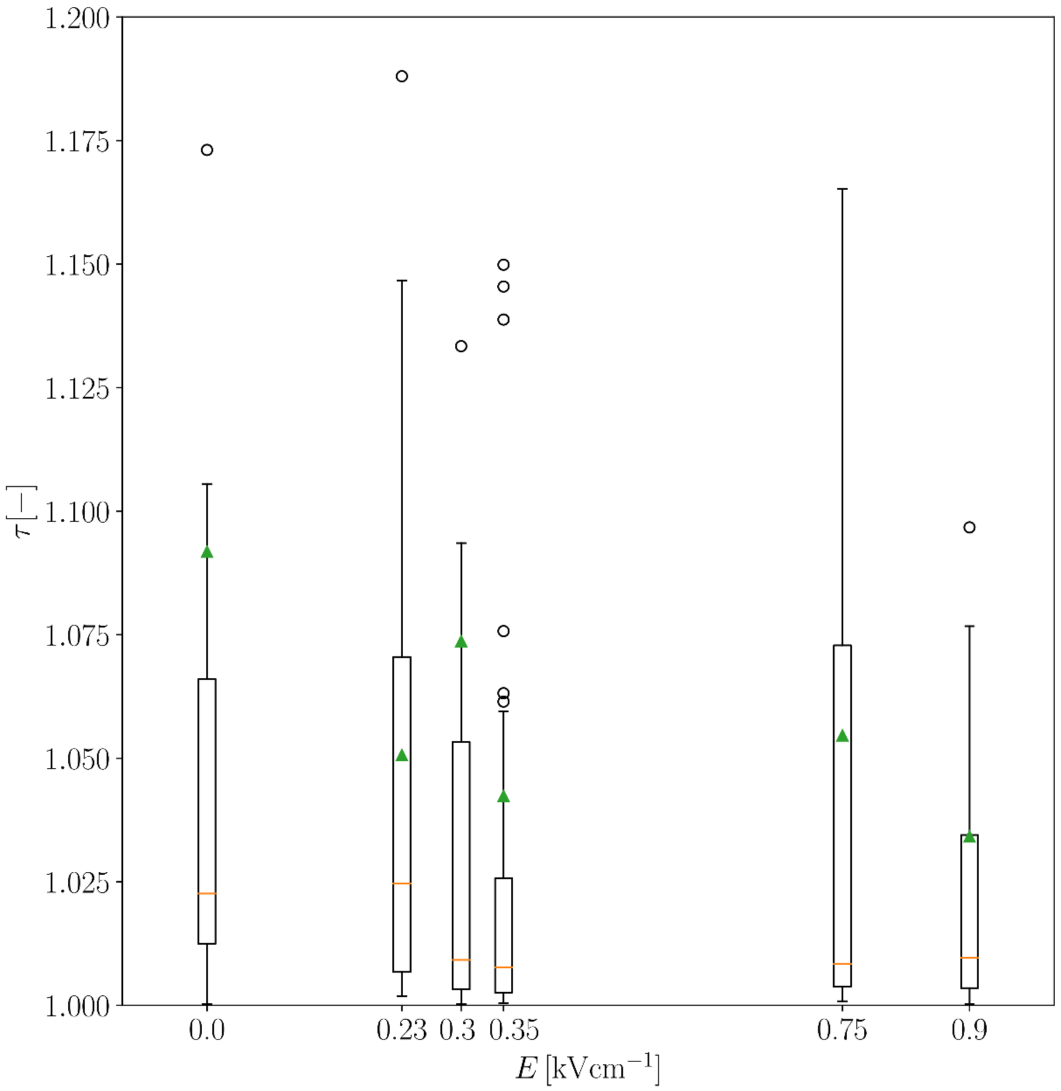

Figure S8. Boxplots of curl ratios measured from SEM images at different field intensities. Whiskers are placed at 1.5 times the interquartile range. Orange lines indicate medians, and green triangles show means.

Waviness Image Analysis

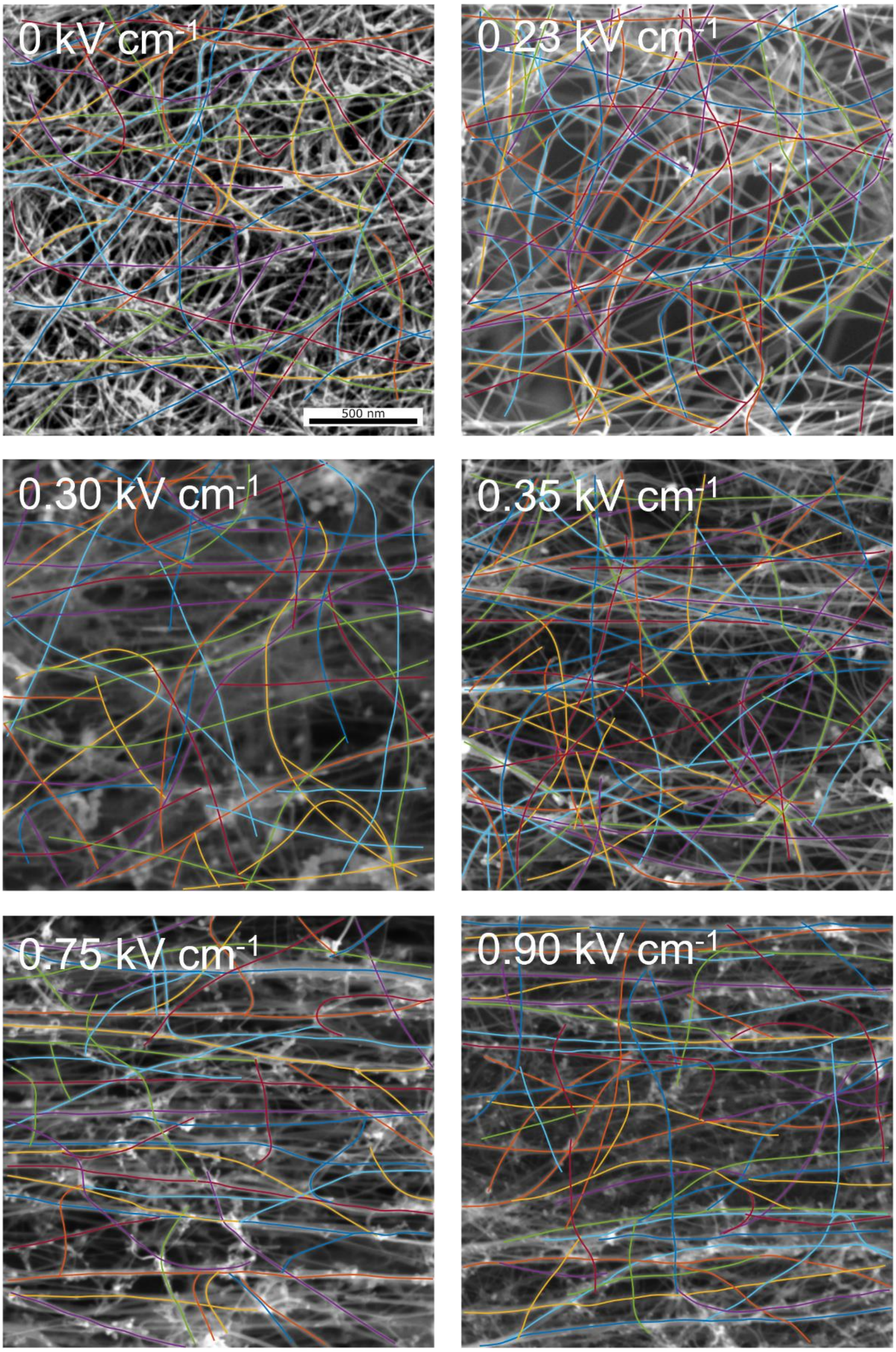

Figure S9. SEM images of CNT materials produced at different field intensities. Colored lines show distinct traced CNT bundles for waviness calculations. Scale bar is shared between images.

Modeling of CNT Alignment Under the Influence of an RF Field

RF current model

The RF model for SWCNTs, and thus the results of this section, are based on the transmission line description of a Luttinger liquid for electrons near the Fermi level by Burke.[1] In the Luttinger liquid, the four conducting states at the Fermi level split into four separate modes, only one of which carries a charge. The charge-carrying mode can be described by a transmission line model shown in Fig. S10 with distributed capacitance $\mathcal{C}$, inductance $\mathcal{L}$, and resistance $\mathcal{R}$ per unit length.

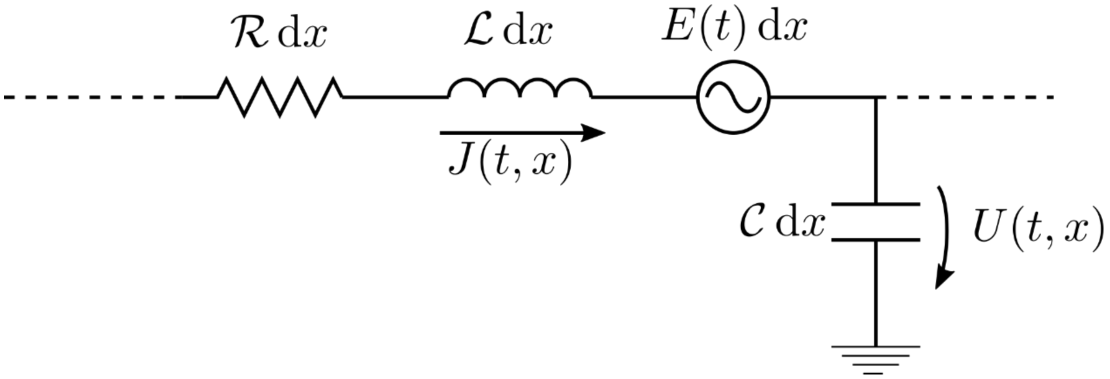

Figure S10. Distributed element model for charge mode in SWCNTs with linear densities for resistance, inductance, and capacitance with the extension of an electric field.

We also note that the model does not take current saturation explicitly into account. In practice, the current in the CNT cannot exceed the saturation current J_0. For simplicity, we will assume that the current simply does not increase further once it reaches J_0 according to the model. A model where current saturation is fully taken into account via a modified resistance term should be considered in future work.

The equations governing the transmission line model are known as the telegrapher's equations, and with the extension of an AC voltage source are given by:

$$\frac{\partial J(t,x)}{\partial x} = -\mathcal{C}\frac{\partial U(t,x)}{\partial t},$$

$$\frac{\partial U(t,x)}{\partial x} = -\mathcal{L}\frac{\partial J(t,x)}{\partial t} - \mathcal{R}J(t,x) + E(t).$$

The capacitance and inductance per unit length are parametrized based on the linear dispersion relation of electrons in a SWCNT near the Fermi level and follow from the finite energy spacing and electronic kinetic energy, respectively:

$$\mathcal{L} = \frac{h}{8e^2 v_\mathrm{F}}, \mathcal{C} = \frac{8e^2 g^2}{h v_\mathrm{F}},$$

where e and h denote the elementary charge and Planck's constant, $g \approx 0.25$ is the electronic interaction factor and $v_{\mathrm{F}} \approx 8.0 \times 10^5$ m s^{-1} is the Fermi velocity for SWCNTs.[1]

The two telegrapher's equations can now be combined into a single second-order PDE for the current:

$$\frac{1}{\mathcal{C}}\frac{\partial^2 J(t,x)}{\partial x^2} = \mathcal{L}\frac{\partial^2 J(t,x)}{\partial t^2} + \mathcal{R}\frac{\partial J(t,x)}{\partial t} - \frac{\partial E(t)}{\partial t}.$$

To find the steady-state solution, we calculate the Fourier transform of the PDE in time, and write in terms of the Fourier transforms of the current $\mathcal{J}(x,\omega)$ and the electric field $\mathcal{E}(\omega)$:

$$\frac{1}{\mathcal{C}}\frac{\partial^2 \mathcal{J}(\omega,x)}{\partial x^2} = -\mathcal{L}\omega^2\mathcal{J}(\omega,x) + i\omega\mathcal{R}\mathcal{J}(\omega,t) - i\omega\mathcal{E}(\omega).$$

Solving this ODE with respect to x with boundary conditions $\mathcal{J}(\omega,0) = \mathcal{J}(\omega,L) = 0$ yields:

$$\mathcal{J}(\omega,x) = \mathcal{E}(\omega)\frac{\left[1 - \cosh\left(\frac{1}{2}\gamma(\omega)(L-2x)\right)\operatorname{sech}\left(\frac{1}{2}\gamma(\omega)L\right)\right]}{\mathcal{R} + i\omega\mathcal{L}},$$

where $\gamma(\omega)$ denotes the propagation constant of the charge carrying mode:

$$\gamma(\omega) = \sqrt{(i\omega\mathcal{C})(\mathcal{R} + i\omega\mathcal{L})}.$$

The AC electric field of angular frequency ω_0 and amplitude E_0 is $E(t) = E_0 \sin(\omega_0 t)$. Then, taking the inverse Fourier transforms yields the steady-state solution of the PDE for the current, after all transient solutions have decayed:

$$J(t,x) = -E_0 \operatorname{Im}\left\{\frac{\left[1 - \cosh\left(\frac{1}{2}\gamma(\omega_0)(L-2x)\right)\operatorname{sech}\left(\frac{1}{2}\gamma(\omega_0)L\right)\right]}{\mathcal{R} + i\omega_0\mathcal{L}}\right\}.$$

This solution shows resonant behavior at the following frequencies:

$$f_n^* = \frac{v_{\mathrm{F}}}{2gL}(1 + 2n).$$

Fig. S11a shows that for CNT lengths of up to the millimeter regime, the resonant frequency is significantly larger than the applied frequency of 13.56 MHz.

In this regime, i.e., for ω_0 “ $2\,\pi\,f_0^*$, the current expression can be expanded to in ω_0:

$$J(t,x) = \frac{1}{2}E_0\mathcal{C}x(L-x)\omega_0 + \mathcal{O}(\omega_0^3).$$

Notice that there is no explicit time dependence of the current in that regime, and the current changes sign every half period of the AC field. Hence, we may assume the magnitude of the current is constant

in time $J(t,x) = J(x)$. Furthermore, the current is a quadratic function with its extremum at the center of the CNT. In this approximation, the average magnitude of the current along the CNT and in time is:

$$\langle |J| \rangle = \frac{1}{L}\int_0^L \mathrm{d}\,x\,J(x) = \frac{1}{12}\mathcal{C}E_0 L^2 \omega_0.$$

Using the same approximation, we can also calculate the length of the CNT for which the current exceeds the saturation current Δ L by solving $J(x) = J_0$ for x. Relative to the length of the CNT L it is:

$$\frac{\Delta L}{L} = \sqrt{1 - \frac{8J_0}{E_0 \mathcal{C} \omega_0 L^2}}.$$

Figures S11b-c show the average current in the CNT, as well as the relative length of the CNT for which the current exceeds the saturation current, as a function of CNT length. In both cases, $f = 13.56$ MHz and E_0=10.0 kV cm^{-1} were chosen to reflect conditions in our experimental setup close to the dielectric breakdown of the hydrogen gas. Both graphs show that for CNT lengths exceeding 300 μm, a significant portion of the CNT length reaches current saturation.

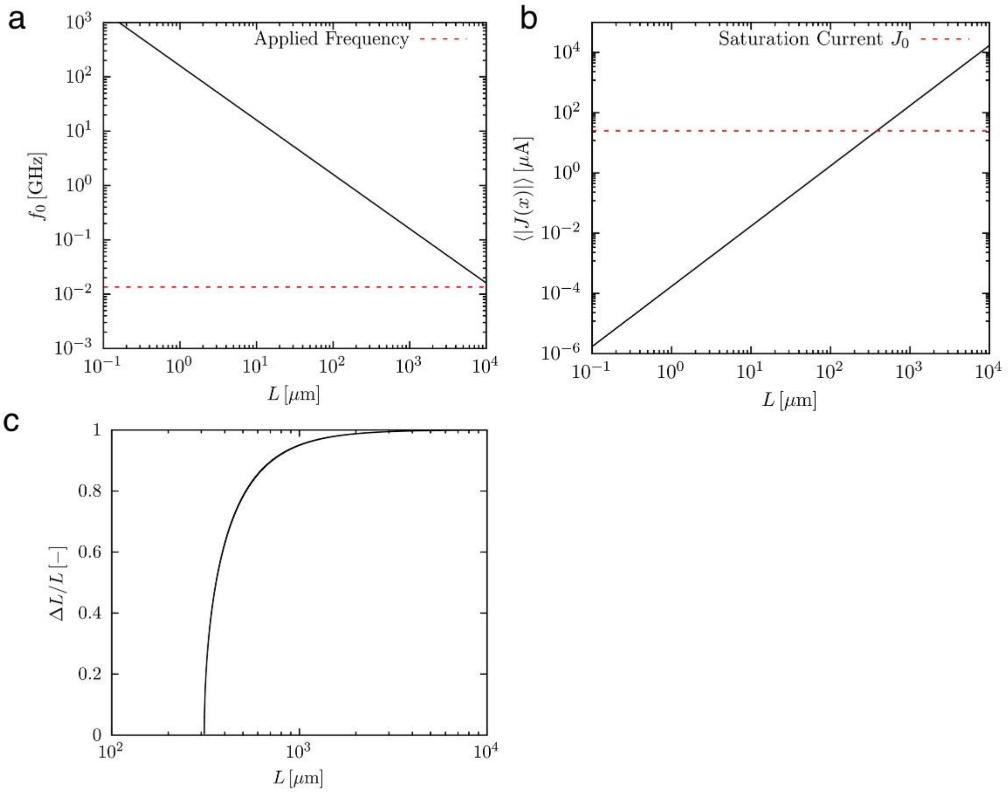

Figure S11. Solutions for transmission line model. (a) Log-log plot of resonance frequency vs CNT length with experimentally applied frequency as a dashed red line. (b) Log-log plot of average current in CNT vs. CNT length with saturation current as dashed red line for reference. (c) Lin-log plot of length proportion at which saturation current is reached vs. CNT length.

Statistical model

The CNT alignment with alternating electric fields can be described using the worm-like chain model with energy contributions from bending, electric polarization, and the additional electromagnetic interactions due to the z-pinch stiffening effect.

As justified in the main text, we will neglect any variation of the current magnitude in the CNT along its contour and in time. Without loss of generality, we fix the direction of the current to point along the tangent vector of the chain $\mathbf{J}(s) = J\hat{\mathbf{t}}(s)$. Here, we derive the free energy contribution of the z-pinch stiffening effect.

From current to pressure

Suppose a current $\mathbf{J}(s) = J\hat{\mathbf{t}}(s)$ is applied to the CNT. In this work, we will assume that a CNT can be modeled as a thin cylindrical shell with internal and external radii R_1, R_2. Assuming a uniform distribution of the current, the current density is given by:

$$\boldsymbol{j}(s) = \frac{J}{\pi(R_2^2 - R_1^2)}\hat{\boldsymbol{t}}(s).$$

In the following, we will assume all electric and magnetic fields are quasi-static such that we can neglect all dynamical terms in the field equations. We introduce a set of cylindrical coordinates that follows the chain contour (r, ϕ, s) with corresponding coordinate unit vectors $\{\hat{\mathbf{r}}(\phi,\ s), \hat{\boldsymbol{\phi}}(\phi,\ s), \hat{\mathbf{t}}(s)\}$. In these coordinates, the resulting magnetic field can be computed using Ampère's law:

$$\boldsymbol{B}(r, \phi, s) = \frac{\mu_0 J(r^2 - R_1^2)}{2\pi(R_2^2 - R_1^2)r}\hat{\boldsymbol{\phi}}(\phi, s).$$

In turn, the current experiences a Lorentz force due to the magnetic field, which is given by:

$$\boldsymbol{f}(r, \phi, s) = \boldsymbol{j}(s) \times \boldsymbol{B}(r, \phi, s) = -\frac{\mu_0 J^2(r^2 - R_1^2)}{2\pi^2 r(R_2^2 - R_1^2)^2}\hat{\boldsymbol{r}}(\phi, s).$$

Note that the force is pointing towards the center of the CNT and should result in compression in a mechanism analogous to the compression and confinement of a plasma using a z-pinch.

As the extension of π-orbitals outside the surface can be considered small compared to the radius of the CNT, we can compute the mean pressure acting on the surface and take the limit of vanishing thickness:

$$\boldsymbol{p}(\phi, s) = \lim_{\substack{R_1 \to R \\ R_2 \to R}} \frac{1}{R}\int_{R_1}^{R_2} \mathrm{d}r\, r\, \boldsymbol{f}(r, \phi, s) = \lim_{\substack{R_1 \to R \\ R_2 \to R}} -\frac{\mu_0 J^2(2R_1 + R_2)}{6\pi^2 R(R_1 + R_2)^2}\hat{\boldsymbol{r}}(\phi, s) = -p\hat{\boldsymbol{r}}(\phi, s),$$

where R is assumed to be the mean radius of the CNT and the pressure acting on the surface of the CNT is given by:

$$p = \frac{\mu_0 J^2}{8\pi^2 R^2}.$$

Note that by taking this limit, the initial assumption of finite wall thickness disappears. Any finite current distribution in the CNT will lead to the same result for the pressure.

From pressure to force

As the WLC model describes an object that is inherently one-dimensional, we need to derive a contour force density from the pressure acting on the shell of the CNT. Let us first parametrize a point $\mathbf{x}(\phi, s)$ on the surface of the CNT in cylindrical coordinates:

$$\boldsymbol{x}(\phi, s) = \boldsymbol{\gamma}(s) + R\hat{\boldsymbol{r}}(\phi, s).$$

Here, $\boldsymbol{\gamma}(s)$ is the position vector of the CNT contour and $\hat{\boldsymbol{r}}(\phi, s)$ is the radial vector perpendicular to the tangent vector at $\boldsymbol{\gamma}(s)$. Next, we define the Frenet-Serret basis $\{\hat{\mathbf{t}}(s), \hat{\mathbf{n}}(s), \hat{\mathbf{b}}(s)\}$ of the chain:

$$\hat{\mathbf{t}}(s) = \frac{\mathrm{d}\boldsymbol{\gamma}(s)}{\mathrm{d}s}, \quad \frac{\mathrm{d}\hat{\boldsymbol{t}}(s)}{\mathrm{d}s} = \kappa(s)\hat{\boldsymbol{n}}(s), \quad \hat{\boldsymbol{b}}(s) = \hat{\boldsymbol{t}}(s) \times \hat{\boldsymbol{n}}(s),$$

where $\kappa(s)$ is the local curvature of the chain. In this basis, the radial vector can be expanded as:

$$\hat{\boldsymbol{r}}(\phi, s) = \cos\phi\,\hat{\boldsymbol{n}} + \sin\phi\,\hat{\boldsymbol{b}}.$$

With this parameterization, we can compute the differential surface element of the CNT:

$$\mathrm{d}A = \left\| \frac{\mathrm{d}\boldsymbol{x}(\phi, s)}{\mathrm{d}\phi} \times \frac{\mathrm{d}\boldsymbol{x}(\phi, s)}{\mathrm{d}s} \right\| \mathrm{d}\phi\, \mathrm{d}s = R(1 - R\kappa(s)\cos\phi)\,\mathrm{d}\phi\,\mathrm{d}s.$$

The line force density is then obtained by integrating out the angular dependence of the radial force:

$$\boldsymbol{q}(s) = \int_0^{2\pi} \mathrm{d}\,\phi\, R(1 - R\kappa(s)\cos\phi)\boldsymbol{p}(\phi, s) = pA\frac{\mathrm{d}\hat{\boldsymbol{t}}(s)}{\mathrm{d}s},$$

where $A = \pi R^2$ is the cross-sectional area of the CNT. Hence, the pressure resulting from the current will always work against the curvature of the chain.

From force to energy

In the next step, we want to derive a free energy expression that will result in the correct force density that we have derived in the above section upon minimization. In order to fully specify one configuration of the chain, we will provide values for the tangent vectors at both ends of the chain $\hat{\mathbf{t}}(0), \hat{\mathbf{t}}(L)$ as well the position of some point $\boldsymbol{\gamma}(s^*)$ along the chain. We will later show that the choice of s^* is unique rather than arbitrary. Further note that the variation of these quantities vanishes by definition. The virtual work done by the force density is given by:

$$\delta W_{\boldsymbol{q}} = pA\int_0^L \mathrm{d}s\,\frac{\mathrm{d}\hat{\boldsymbol{t}}(s)}{\mathrm{d}s}\cdot\delta\boldsymbol{\gamma}(s) = pA\big(\hat{\boldsymbol{t}}(L)\cdot\delta\boldsymbol{\gamma}(L) - \hat{\boldsymbol{t}}(0)\cdot\delta\boldsymbol{\gamma}(0)\big) - pA\int_0^L \mathrm{d}s\,\hat{\boldsymbol{t}}(s)\cdot\delta\hat{\boldsymbol{t}}(s),$$

where we have integrated by parts. The remaining integral vanishes since $2\,\hat{\mathbf{t}}(s)\cdot\delta\hat{\mathbf{t}}(s) = \delta(\hat{\mathbf{t}}(s)^2) = \delta(1) = 0$.

The resulting expression has the natural interpretation of a chain that is being stretched by two forces of magnitude pA pulling in the direction of both ends.

As the variation of the specified position $\delta\boldsymbol{\gamma}(s^*) = 0$, we may write for the virtual work:

$$\begin{aligned}\delta W_{\mathbf{q}} &= pA\big[\hat{\mathbf{t}}(L)\cdot\delta\big(\boldsymbol{\gamma}(L)-\boldsymbol{\gamma}(s^*)\big) - \hat{\mathbf{t}}(0)\cdot\delta\big(\boldsymbol{\gamma}(0)-\boldsymbol{\gamma}(s^*)\big)\big]\\ &= pA\,\delta\left[\int_0^{s^*}\mathrm{d}s\,\hat{\mathbf{t}}(0)\cdot\hat{\mathbf{t}}(s) + \int_{s^*}^{L}\mathrm{d}s\,\hat{\mathbf{t}}(L)\cdot\hat{\mathbf{t}}(s)\right].\end{aligned}$$

The variation of the potential of the force density is simply $\delta F_{\mathbf{q}} = -\delta W_{\mathbf{q}}$, such that we obtain the following expression for the potential:

$$F_{\mathbf{q}} = -pA\left[\int_0^{s^*}\mathrm{d}s\,\hat{\mathbf{t}}(0)\cdot\hat{\mathbf{t}}(s) + \int_{s^*}^{L}\mathrm{d}s\,\hat{\mathbf{t}}(L)\cdot\hat{\mathbf{t}}(s)\right],$$

up to a constant. Finally, we require that the energy is invariant under inversion of the chain $\boldsymbol{\gamma}(s) \to \boldsymbol{\gamma}(L-s)$, as there should be no preferred direction of the chain. Under this symmetry, the tangent vector transforms as $\hat{\mathbf{t}}(s) \to -\hat{\mathbf{t}}(L-s)$ and the transformed free energy is:

$$F_{\mathbf{q}} \to -pA\left[\int_0^{L-s^*}\mathrm{d}s\,\hat{\mathbf{t}}(0)\cdot\hat{\mathbf{t}}(s) + \int_{L-s^*}^{L}\mathrm{d}s\,\hat{\mathbf{t}}(L)\cdot\hat{\mathbf{t}}(s)\right].$$

By imposing invariance under the given symmetry, we quickly find $s^* = L/2$, such that we can write for the free energy of the internal pressure:

$$F_{\mathbf{q}} = -pA\left[\int_0^{L/2}\mathrm{d}s\,\hat{\mathbf{t}}(0)\cdot\hat{\mathbf{t}}(s) + \int_{L/2}^{L}\mathrm{d}s\,\hat{\mathbf{t}}(L)\cdot\hat{\mathbf{t}}(s)\right].$$

Thus, we have arrived at the main modeling result of this work. Again, the above free energy expression has the natural interpretation of both halves of the chain being pulled in the direction of their respectively closest ends, with the mid-point of the chain being fixed in place.

Model Parametrization

Current, Cross-Sectional Area and Temperature

The current J is taken to be an appropriate multiple of the saturation current J_0 based on the number of CNT walls present in the structure.

The cross-sectional area is determined by the radius of the CNTs. In the case of SWCNT bundles, the sum of the cross-sectional areas of the SWCNTs is used. For MWCNTs, we choose the radius of the outermost CNT.

Temperature is taken into account by statistical averaging over the canonical ensemble of all WLC configurations. In all results presented in this work, the temperature was chosen as $T = 1200$ K, but was generally found to only have a negligible effect on the resulting alignment compared to all other model parameters.

Stiffness Parametrization

In the above free energy expression, we still need to specify the stiffness a of the CNT structure under investigation. For SWCNTs, we use the following empirical formula calculated for armchair CNTs using Molecular Dynamics simulations [2]:

$$a_{\mathrm{SWCNT}} = 63.80\left(R/\text{Å}\right)^{2.93} \text{ eV Å}.$$

Bundles of SWCNTs have different scaling due to the interaction of the π-orbitals given by [3]:

$$a_B = a_{\mathrm{SWCNT}} \left(\frac{R}{R_{\mathrm{B}}}\right)^{1.9}.$$

Finally, the scaling for MWCNTs follows by the addition of the bending moments, leading to the addition of the stiffnesses over the number of CNT walls N_{W} [4]:

$$a_{MWCNT} = \sum_{i=1}^{N_W} a_{\mathrm{SWCNT,i}}.$$

***T*₂ From 3D Model**

The model described above is inherently three-dimensional. The order parameter T_2 however describes alignment in two dimensions. For convenience, we repeat the definition of T_2 from the manuscript:

$$T_2 = \langle \cos^2 \theta_{2\mathrm{D}} \rangle - 1.$$

We, therefore, need to derive the above average in terms of the two-dimensional alignment angle from three dimensions. First, consider a general three-dimensional unit vector in spherical coordinates:

$$\hat{r} = \begin{pmatrix} \cos\phi \sin\theta_{3\mathrm{D}} \\ \sin\phi \sin\theta_{3\mathrm{D}} \\ \cos\theta_{3\mathrm{D}} \end{pmatrix}.$$

The projection of this vector onto the x_2-x_3 plane is given by:

$$r_\perp = \begin{pmatrix} \sin\phi \sin\theta_{3\mathrm{D}} \\ \cos\theta_{3\mathrm{D}} \end{pmatrix}.$$

From this, the cosine of the two-dimensional alignment angle with the x_3 axis in the x_2-x_3 plane θ_{2D} is:

$$\cos\theta_{2D} = \frac{r_3}{\sqrt{r_2^2 + r_3^2}} = \frac{\cos\theta_{3D}}{\sqrt{\cos^2\theta_{3D} + \sin^2\theta_{3D}\sin^2\phi}}.$$

Assuming the 3D orientation distribution function of the system is a function θ_{3D}, we may calculate the mean value of the squared cosine of the two-dimensional alignment angle θ_{2D} as follows:

$$\langle\cos^2\theta_{2D}\rangle = \frac{1}{2\pi}\int_0^{\pi} \mathrm{r}d\,\theta_{3D}\int_0^{2\pi} \mathrm{d}\,\phi\ \sin\theta_{3D}\,f(\theta_{3D})\frac{\cos^2\theta_{3D}}{\cos^2\theta_{3D} + \sin^2\theta_{3D}\sin^2\phi}.$$

Evaluating the integral in ϕ then yields:

$$\langle\cos^2\theta_{2D}\rangle = \frac{1}{2\pi}\int_0^{\pi} \mathrm{r}d\,\theta_{3D}\int_0^{2\pi} \mathrm{d}\,\phi\ \sin\theta_{3D}\,f(\theta_{3D})\,|\cos\theta_{3D}| = \langle|\cos\theta_{3D}|\rangle.$$

In the well-aligned regime, we have $\langle|\cos\theta_{3D}|\rangle. \approx \langle\cos\theta_{3D}\rangle$, where we document how to calculate the latter average in the harmonic approximation in our previous work, to which the reader is referred to for details. [5]

References

(1) Burke, P. J. Luttinger Liquid Theory as a Model of the Gigahertz Electrical Properties of Carbon Nanotubes. *IEEE Trans. Nanotechnol.* **2002**, *1* (3), 129–144.

(2) Zhigilei, L. V; Wei, C.; Srivastava, D. Mesoscopic Model for Dynamic Simulations of Carbon Nanotubes. *Phys. Rev. B* **2005**, *71* (16), 165417.

(3) Adnan, M.; Pinnick, R. A.; Tang, Z.; Taylor, L. W.; Pamulapati, S. S.; Carfagni, G. R.; Pasquali, M. Bending Behavior of CNT Fibers and Their Scaling Laws. *Soft Matter* **2018**, *14* (41), 8284–8292.

(4) Pantano, A.; Parks, D. M.; Boyce, M. C. Mechanics of Deformation of Single-and Multi-Wall Carbon Nanotubes. *J. Mech. Phys. Solids* **2004**, *52* (4), 789–821.

(5) Kloza, P. A.; Elliott, J. A. Freely Suspended Semiflexible Chains in a Strong Aligning Field: Simple Closed-Form Solutions for the Small-Angle Approximation. *Macromol. Theory Simulations* **2020**, *29* (6), 2000049. https://doi.org/10.1002/MATS.202000049.
